# Tongxie Yaofang ameliorates IBS-D by targeting the gut microbiota-derived tryptophan metabolites and AhR signaling axis

**DOI:** 10.3389/fmicb.2026.1786701

**Published:** 2026-04-30

**Authors:** Xiangyu Xie, Weixin Yan, Chunli Gan, Wei Xiong, Yusheng Huang, Lijun Lin, Xiaochun Lei, Wei Ke, Yuna Chai, Hongmei Tang, He Zhu

**Affiliations:** 1 The First Clinical School of Guangzhou University of Chinese Medicine, Guangzhou, China; 2 The First Affiliated Hospital of Guangzhou University of Chinese Medicine, Guangzhou, China; 3 Lingnan Research Center of Guangzhou University of Chinese Medicine, Guangzhou, China; 4 Guangdong Clinical Research Academy of Chinese Medicine, Guangzhou, China; 5 Zhongshan Hospital of Traditional Chinese Medicine, Zhongshan, China; 6 Foshan Hospital of Traditional Chinese Medicine, Foshan, China; 7 The First Affiliated Hospital of ZhengZhou University, Zhengzhou, China

**Keywords:** Tongxie Yaofang (TXYF), IBS-D, gut microbiota, tryptophan, intestinal barrier, aryl hydrocarbon receptor

## Abstract

**Background and aims:**

Tongxie Yaofang (TXYF) is a classic prescription for IBS-D with liver depression and spleen deficiency, with its therapeutic mechanisms requiring further elucidation. This study investigated the modulatory effects of TXYF on the gut microbiota and microbiota-derived metabolism in an IBS-D rat model to elucidate the underlying mechanisms.

**Methods:**

HPLC was employed to identify the main components of TXYF. An IBS-D rat model was replicated using a triple-factor approach that combined neonatal maternal separation, chronic restraint stress, and oral gavage of *Sennae folium* decoction. To elucidate the mechanisms underlying the effects of TXYF on IBS-D, the gut microbiota was assessed by 16S rRNA sequencing, and microbial metabolites were profiled via untargeted metabolomics. Furthermore, key regulatory factors were examined by immunohistochemistry (IHC), reverse transcription-quantitative polymerase chain reaction (RT-qPCR), and western blotting. Finally, fecal microbiota transplantation (FMT) was performed to validate the pathogenic role of the microbiota and the therapeutic potential of TXYF.

**Results:**

TXYF significantly alleviated IBS-D symptoms in rats, including diarrhea and abdominal pain, and improved both depressive-like behavior and intestinal barrier function. Treatment with TXYF increased the abundance of *Bifidobacterium* in the gut microbiota and promoted the microbial-related metabolic conversion of tryptophan (TRP) to 5-hydroxyindoleacetic acid (5-HIAA), indole-3-acetic acid (IAA), tryptamine, 5-hydroxytryptamine (5-HT), and 2-oxindole. These metabolites activated the aryl hydrocarbon receptor (AhR) signaling pathway, thereby inhibiting MLC phosphorylation and decreasing MLCK expression, and ultimately restoring intestinal barrier function. Furthermore, the FMT experiment demonstrated that the microbiota from TXYF-treated rats significantly ameliorated IBS-D by activating the AhR signaling pathway.

**Conclusion:**

TXYF may alleviate IBS-D symptoms and restore barrier function by increasing the abundance of *Bifidobacterium*, restoring tryptophan metabolism, and activating the AhR pathway.

## Graphical Abstract

**Figure:**
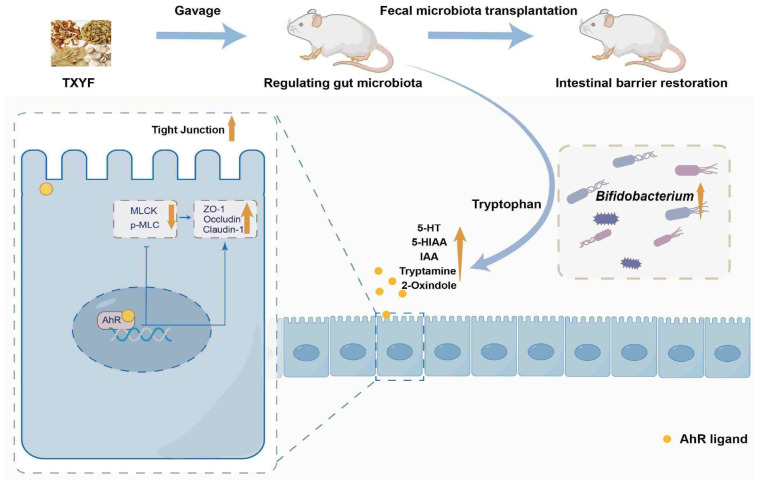
TXYF alleviates IBS-D symptoms and restores intestinal barrier function by enriching *Bifidobacterium*, promoting TRP metabolism (5-HT, 5-HIAA, IAA, tryptamine, 2-oxindole), and activating the AhR signaling pathway to inhibit MLCK/p-MLC and upregulate tight junction proteins (ZO-1, Occludin, Claudin-1). The FMT experiment demonstrated that the microbiota from TXYF-treated donors recapitulated these protective effects.

## 1 Introduction

Irritable bowel syndrome (IBS), characterized by altered bowel habits and abdominal pain, has a global prevalence of approximately 4–10% and is increasing (Greer and Sultan, 2025). Notably, the prevalence among high-stress groups such as medical students can reach up to 30% (Zhang et al., 2016; Liu et al., 2014). The high prevalence and complex, heterogeneous clinical presentation of IBS contribute to its substantial burden on both individuals and public health systems.

Although the exact etiology of IBS remains unclear, its pathogenesis is generally attributed to intestinal barrier dysfunction, gut microbiota imbalance, and low-grade inflammation, with variations across subtypes (Ford et al., 2020). Among these, diarrhea-predominant IBS (IBS-D), characterized by recurrent diarrhea, is the most prevalent subtype in China (Liu and Liu, 2021). In patients with IBS-D, the function and expression of tight junction (TJ) proteins are impaired. Restoring TJ protein function can alleviate the symptoms of IBS-D (Bertiaux-Vandaele et al., 2011). Altered microbiome and reprogrammed metabolome provide clues in regulating the intestinal epithelial barrier—the body’s largest mucosal surface (Barbara et al., 2016; Edogawa et al., 2020). Normally, the gut microbiota and the host intestine exist in a state of mutualism that is essential for sustaining a stable and balanced internal environment (Li et al., 2016). While IBS-D patients exist gut dysbiosis, manifested as decreased microbial diversity (Pittayanon et al., 2019) and an imbalance in community composition—specifically, an increase in *Ruminococcus* and *Fusobacterium* (Zhou et al., 2016; Zhai et al., 2023) and a decrease in *Lactobacillus* and *Bifidobacterium* (Pittayanon et al., 2019). This dysbiosis is also associated with alterations in bacterial metabolites, such as short-chain fatty acids (SCFAs), bile acids, tryptophan (TRP), and its derivatives, which in turn affect mucosal integrity and intestinal homeostasis (Wang et al., 2023; Han et al., 2022; Yu et al., 2018). These findings suggest the therapeutic potential of restoring intestinal barrier integrity by targeting specific microbial metabolites in IBS-D.

The multi-component, multi-target profile of Traditional Chinese Medicine (TCM) enables it to act as a modulator of the gut microbiota, exerting effects both directly on microbial communities and indirectly via host-mediated pathways (Zhang S. et al., 2024; Wei et al., 2024; Qian et al., 2023). Tongxie Yaofang (TXYF) is one of the classic TCM formulations for treating IBS-D, known for its effects in soothing the liver, alleviating depression, and relieving pain. It is composed of four herbal ingredients: *Atractylodis Macrocephalae Rhizoma* (Bai Zhu), *Paeoniae Radix Alba* (Bai Shao), *Citri Reticulatae Pericarpium* (Chen Pi), and *Saposhnik Oviae Radix* (Fang Feng), in a ratio of 9:6:3:1. Although previous research has demonstrated that TXYF regulates TJ proteins, immune environment, neurotrophic factors, and the intestinal mucosal microbiota (Lu and Zhang, 2023; Long et al., 2025; Shang et al., 2025), the mechanisms underlying its microbiota-mediated benefits, particularly regarding the improvement of intestinal barrier function, are not yet fully understood.

To elucidate the mechanisms of TXYF in IBS-D, we first profiled the gut microbiota and metabolites in IBS-D model rats using 16S rRNA sequencing and untargeted metabolomics. Aryl hydrocarbon receptor (AhR) signaling pathway was identified as a pivotal axis activated by TXYF. Moreover, we validated the functional role of the TXYF-reshaped microbiota through fecal microbiota transplantation (FMT) experiments. Collectively, our study demonstrated how TXYF treats IBS-D through microbiota and metabolite remodeling, providing novel evidence for TCM targeting the gut ecosystem for IBS-D therapy.

## 2 Materials and methods

### 2.1 Chemicals and reagents

*Atractylodis Macrocephalae Rhizoma* (batch number: 2411052) and *Citri Reticulatae Pericarpium* (batch number: 2411128) were purchased from Guangdong Zihetang Pharmacy Co., Ltd. (Guangzhou, China). White *Paeoniae Radix Alba* (batch number: B4174111) was purchased from Guangdong Medicinal Materials Company (Guangzhou, China). *Saposhnik Oviae Radix* (batch number: 241101) and *Sennae folium* formula granules were purchased from Guangdong Yifang Pharmaceutical Co., Ltd. (Foshan, China). The herbal materials were authenticated by Associate Researcher Yusheng Huang (The First Affiliated Hospital of Guangzhou University of Chinese Medicine) according to the Chinese Pharmacopoeia (2020 edition), and the voucher specimens have been deposited at the same institution. Ampicillin (item number: A830931), Neomycin sulfate (item number: N814740), vancomycin hydrochloride (item number: V820413), metronidazole (item number: M813526), and methanol (HPLC grade, item number: M813907) are all from Shanghai Macklin Biochemical Co., Ltd.

### 2.2 Preparation of TXYF and *Sennae folium* decoction

A 45 g of *Citri Reticulatae Pericarpium*, 90 g of *Atractylodis Macrocephalae Rhizoma*, 60 g of *Paeoniae Radix Alba*, and 30 g of *Saposhnik Oviae Radix* were decocted with 2250 mL of water for 1 hour, then filtered. An additional 1800 mL of water was added to decoct the medicinal residue for another 1 hour, followed by filtering again. The combined filtrates were concentrated to a crude drug dosage of 0.82 g/mL. TXYF was stored at 4°C and diluted with sterile distilled water to achieve the target administration concentration. The same method was used to prepare water extracts of different herbs for the identification of fingerprint peaks.

*Sennae folium* formula granules (200 g) were dissolved in boiling water to prepare a medicinal solution containing 0.5 g/mL of crude drug.

### 2.3 HPLC analysis for TXYF

An appropriate amount of methanol was added to the concentrated solution of TXYF and sonicated for 20 min to prepare the test solution with a concentration of 86 mg/mL. Appropriate amounts of reference standards, including paeoniflorin, hesperidin, coumarin glycoside, and 5-methylvisamminol glycoside, were dissolved in methanol and volumetrically adjusted to 5 mL to prepare the mixed reference standard solution.

The Agilent 1260 High-performance liquid chromatography (HPLC) system was used for detection. The Acclaim™120 column (4.6 mm × 250 mm, 5μm) was maintained at 30 °C. The mobile phase consisted of methanol (solvent A) and ultrapure water (solvent B). Samples (10 μL) were injected and separated at a flow rate of 1.0 mL/min, with detection at 230 nm. Detailed methods are provided in Table 1. The composition of TXYF was determined through a comprehensive analysis of retention times.

**TABLE 1:** HPLC analysis parameters employed for TXYF.

Time/min	Proportion of A phase	Proportion of B phase
5	30	70
25	45	55
50	80	20
55	100	0
60	100	0
61	15	85
65	15	85

### 2.4 Animals

Pregnant rats (300–400 g) were obtained from the Guangdong Experimental Animal Center [certification Nos. 44007200127124, 44007200127725; license No. SCXK (Yue) 2022-0002] and housed in an SPF environment with a 12-h light/12-h dark cycle at the Experimental Center of the First Affiliated Hospital of Guangzhou University of Chinese Medicine (Guangzhou, China), following approval by the Hospital’s Ethics Committee (Approval No. GZTCMF1-20230053).

### 2.5 Establishment of IBS-D rat models and treatment

I. Following birth, 40 rats were allocated into the Control group (*n* = 8) and the model group (*n* = 32), with a balanced male-to-female ratio in each group. The neonatal maternal separation began on the third day after birth, lasting for 3 h per day over 14 consecutive days, followed by 3-h daily restraint stress using 3M tape from day 21 to 35. From day 28, *Sennae folium* decoction was administered orally (5 g/kg) for 7 consecutive days. After modeling, the model group was randomly divided into the 4 group (*n* = 8): IBS-D group, Rifaximin group (72 mg/kg), TXYF low dose (TXYF-L, 3.67 g/kg) group, and TXYF high dose (TXYF-H, 7.34 g/kg) group, which were orally administered the respective treatments continuously for 10 days. Rat feces were collected to prepare for subsequent FMT procedures. The feces were homogenized in sterile PBS to achieve a suspension concentration of 200 mg/mL. Subsequently, the homogenized mixture was centrifuged at 4 °C and 10 × g for 30 s. Following this, the supernatant was centrifuged again at 4 °C and 800 × g for 3 min. The resulting precipitate was collected and washed three times with sterile PBS. Finally, FMT samples were prepared by resuspending the washed precipitate in an equal volume of sterile PBS containing 10% glycerol and stored at −80°C.

II. Following birth, the rat pups were allocated into the normal group (*n* = 16) and the model group (*n* = 24), with a balanced male-to-female ratio in each group, and subjected to the modeling protocol described in Part I. On days 30–35, all groups received Antibiotics gavage for 5 days, consisting of Vancomycin hydrochloride (100 mg/kg), Neomycin (200 mg/kg), Metronidazole (200 mg/kg), and Ampicillin (200 mg/kg), to establish the pseudo-germ-free model (Yi et al., 2023). On day 35, the normal animals were assigned to two groups (*n* = 8 per group): the PBS group and the IBS-D-FMT group. The IBS-D-FMT group received fecal microbiota from the IBS-D group in Part I, while the PBS group was administered sterile PBS containing 10% glycerol. The model rats were randomly allocated into three groups (*n* = 8): the IBS-D model group, the TXYF-FMT treatment group, and the Control-FMT group. The TXYF-FMT and Control-FMT groups underwent FMT using feces from the respective TXYF-H and Control groups of Part I. The IBS-D model group received enemas with sterile PBS containing 10% glycerol. All enemas were administered daily at a volume of 1 mL per rat for 10 days.

### 2.6 Evaluation of TXYF efficacy

Post-treatment assessments comprised: fecal moisture content (FMC) determination through fresh fecal sample collection, desiccation at 60°C to constant weight, and calculation using the formula [FMC (%) = 100 × (initial weight − final weight)/initial weight]; visceral pain threshold assessment via the abdominal withdrawal reflex (AWR) scoring system (Ouyang et al., 2024), with colorectal distension volumes recorded when AWR reached score 3 by blinded experimenters; sucrose preference testing (SPT) conducted after 12-h food/water deprivation using a two-bottle choice paradigm (1% sucrose vs. distilled water) with 1-h intake monitoring.

### 2.7 Hematoxylin & Eosin (HE) and Periodic acid-Schiff (PAS) staining

Following sectioning (4 μm), deparaffinization, and rehydration, the paraffin-embedded colon tissue sections were stained with HE and PAS, and then scanned using a digital slide scanner for observation (3D HISTECH, Hungary). The average optical density (AOD) of PAS staining, calculated as the integrated optical density (IOD) divided by the tissue area, was analyzed using ImageJ software.

### 2.8 DNA extraction and 16S rRNA amplification

Extraction and sequencing work were carried out by Novogene Technology Co., Ltd. In short, total genomic DNA was isolated from intestinal contents using a DNA assay kit (TIANamp Soil DNA Kit, TIANGEN, China). The V3-V4 regions of the bacterial 16S rRNA gene were amplified using primers (Forward primer: 5’- CCTAYGGGRBGCASCAG-3’, Reverse primer: 5’- GGACTACNNGGGTATCTAAT-3’) following PCR technology. Library quality assessment was performed using the NovaSeq 6000 System (Illumina, America). Following quality control approval, the NovaSeq PE250 platform (Illumina, America) was utilized for 16S rRNA sequencing. The Novo Cloud platform^1^ was used for data analysis.

### 2.9 Metabolomic analysis

Extraction and sequencing work were carried out by Novogene Technology Co., Ltd. Briefly, intestinal content samples were pulverized in liquid nitrogen, homogenized in ice-cold 80% methanol, and incubated on ice for 5 min. After centrifugation (15,000 × g, 20 min, 4°C), an aliquot of the supernatant was diluted with ultrapure water to 53% methanol, centrifuged again, and the final supernatant was injected into the UHPLC-MS/MS system for analysis. The Novo Cloud platform^1^ was used for data analysis.

### 2.10 ELISA

Following isoflurane anesthesia, arterial blood was collected from the rats. Serum was separated by centrifugation (3,000 rpm, 10 min, 4°C), was stored at −80°C for analysis. The serum levels of diamine oxidase (DAO), TNF-α, and IL-10 were quantified using ELISA kits (Bioswamp, Cat#RA20028, Cat#RA20035, Cat#RA20090) according to the manufacturer’s instructions.

### 2.11 Immunohistochemistry (IHC)

The colon tissue slices were repaired using sodium citrate antigen repair buffer (pH 6.8). After quenching endogenous peroxidase activity with 3% H2O2 and blocking with 5% BSA, slides were incubated overnight at 4°C with the following primary antibodies: ZO-1 (1:400, #AF5145, Affinity), Claudin-1 (1:200, #13050-1-AP, Proteintech), Occludin-1 (1:1,000, #27260-1-AP, Proteintech), AhR (1:300, #67785-1-Ig, Proteintech), and MUC2 (1:1,000, #GB11344-50, Servicebio). After secondary antibody incubation and DAB development, positive cells were identified by brown cytoplasmic staining. Digital images were acquired using the Slice Scanner (3D HISTECH, Hungary). The AOD was analyzed using ImageJ software.

### 2.12 Reverse transcription-quantitative polymerase chain reaction (RT-qPCR)

Total RNA from colon tissue was extracted using TRIzol, and then reverse-transcribed into cDNA (#AG11706, Accurate Biology, China). qPCR was performed using SYBR Green reagent (#AG11702, Accurate Biology, China) on an ABI QuantStudio5 system (Thermo Fisher Scientific, America). Primer sequences (Table 2) were used to quantify relative mRNA expression, normalized to β-actin or GAPDH.

**TABLE 2:** Gene primer sequences for RT-qPCR.

Gene	Forward primer (5’–3’)	Reverse primer (5’–3’)	GenBank accession no.
ZO-1	ATCCCACAAGGAGCCATTCC	TCACAGTGTGGCAAGCGTAG	XM_017588935.3
Claudin-1	CTGTGGATGTCCTGCGTTTC	CCAGCAGGATGCCAATTACC	NM_031699.3
Occludin	CCTTCTTTCCTTAGGCGACC	TTGGTCGAACGTGCATCTCT	NM_031329.3
TNF-α	CGTCCCTCTCATACACTGGC	GCTTGGTGGTTTGCTACGAC	NM_012675.3
IL-10	TTGAACCACCCGGCATCTAC	CCAAGGAGTTGCTCCCGTTA	NM_012854.2
AhR	CACAGCCAGCGGTCTACTAC	CGCTCCGTCTTTCCCTTTCT	NM_001308254.1
CYP1A1	GACAAACACCTGAGTGAGAAGG	GAGAAAGACCTCCAGTCGGC	NM_012540.3
β-actin	GGAGATTACTGCCCTGGCTCCTA	GACTCATCGTACTCCTGCTTGCTG	NM_031144.3
GAPDH	GGCACAGTCAAGGCTGAGAATG	ATGGTGGTGAAGACGCCAGTA	XM_063285519.1

### 2.13 Western blotting

Proteins were extracted from colon tissue, quantified by BCA assay, separated by SDS-PAGE (20 μg/lane), transferred onto PVDF membranes, and probed overnight at 4°C with the following antibodies: ZO-1 (1:15,00, #AF5145, Affinity); Claudin-1 (1:1,000, #13050-1-AP, Proteintech); Occludin-1 (1:5,000, #27260-1-AP, Proteintech); AhR (1:1,500, #67785-1Ig, Proteintech), CYP1A1 (1:2,000, #13241-1-AP, Proteintech); MLCK (1:2,000, #YT2942, Immunoway); p-MLC (1:1,000, #YM8561, Immunoway). Subsequently, the membranes were incubated with the respective secondary antibody at room temperature for 1 h. Signal intensity was determined using the ChemiDoc MP system (Bio-Rad, America) and quantified using Image J software.

### 2.14 Statistical analysis

Data were analyzed using GraphPad Prism 8.0 and are presented as mean ± standard deviation (SD). Multiple comparisons were performed using one-way ANOVA followed by Dunnett’s T3 test. Statistical significance was set at *p-*value < 0.05, *p < 0.05, **p < 0.01; #*p* < 0.05, ##*p* < 0.01.

## 3 Results

### 3.1 HPLC chromatogram of TXYF

**FIGURE 1:**
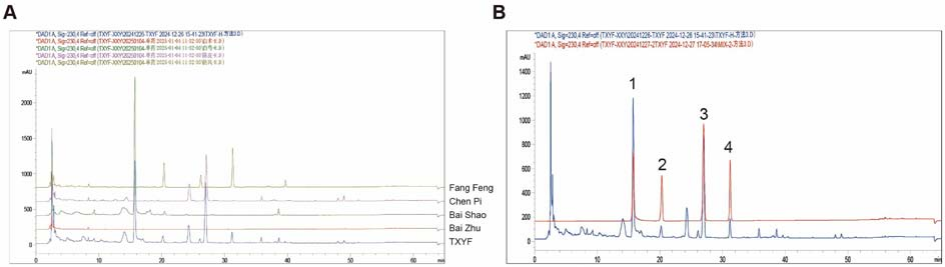
HPLC chromatogram of TXYF. **(A)** Attribution of chromatographic peaks in the TXYF fingerprint. **(B)** Identification of paeoniflorin (1), prim-O-glucosylcimifugin (2), hesperidin (3), and 5-O-methylvisammioside (4) in TXYF.

The representative HPLC chromatographic profile of the TXYF extract is shown in Figure 1. Compounds were identified by comparing their retention times and UV spectra with individual herbal extracts and standards under identical analytical conditions. Peaks 1–4 were identified as paeoniflorin, prim-O-glucosylcimifugin, hesperidin, and 5-O-methylvisammioside.

### 3.2 TXYF demonstrated therapeutic efficacy against IBS-D symptoms

**FIGURE 2:**
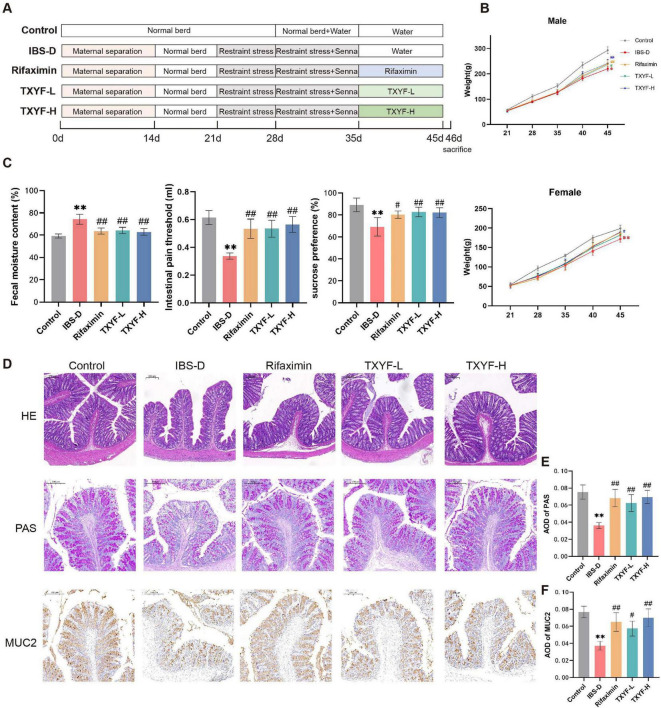
TXYF alleviated symptoms in IBS-D rats. **(A)** Schematic of the experimental IBS-D model with liver depression and spleen deficiency. **(B)** Body weight in each group (male rats, *n* = 4; female rats, *n* = 4). **(C)** Fecal moisture content (*n* = 8), intestinal pain threshold (n = 8), and sucrose preference (*n* = 6) in each group. **(D)** Representative images of HE staining (100 × ), PAS staining (150 × ), and MUC2 immunohistochemistry (150 × ) in the colon. **(E,F)** Quantification of PAS, MUC2 AOD in the colon (*n* = 5). Values are expressed as the mean ± SD. Compared with the Control group, *p < 0.05, **p < 0.01; compared with the IBS-D group, #*p* < 0.05, ##*p* < 0.01.

An IBS-D rat model was induced via maternal separation, restraint stress, and *Sennae folium* decoction (Figure 2A). Following successful model induction, the experimental rats demonstrated decreased locomotor activity, heightened irritability, impaired growth rate, and persistent diarrhea. After administration, the symptoms of IBS-D in each treatment group improved to some extent. Compared with the IBS-D group, both TXYF-L and TXYF-H groups showed significant improvements in weight, FMC, and threshold for intestinal pain, as well as a significant increase in sucrose preference (Figures 2B,C). Notably, the TXYF-H group demonstrated superior therapeutic efficacy compared with the TXYF-L group, with effects comparable to those achieved by the Rifaximin group.

HE staining revealed no significant pathological damage in the colon of any group (Figure 2D). The intestinal mucosal barrier, a critical component of the gut barrier system, was evaluated through PAS staining and IHC to assess goblet cell density and MUC2 expression patterns (Figures 2D–F). In IBS-D model animals, goblet cells responsible for mucus secretion were absent and damaged, and the levels of MUC2 protein were reduced. Treatment with Rifaximin and TXYF reversed these changes.

### 3.3 TXYF ameliorated intestinal hyperpermeability and low-grade inflammation in IBS-D rats

**FIGURE 3:**
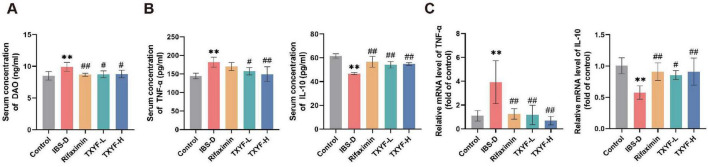
TXYF ameliorated intestinal permeability and inflammatory response in IBS-D rats. **(A)** Serum concentration of DAO in each group (*n* = 6). **(B)** Serum concentrations of TNF-α, IL-10 in each group (*n* = 6). **(C)** Relative mRNA levels of TNF-α and IL-10 in the colon (*n* = 6). Values are expressed as the mean ± SD. Compared with the Control group, **p < 0.01; compared with the IBS-D group, #*p* < 0.05, ##*p* < 0.01.

Upon intestinal mucosal injury, substantial amounts of diamine oxidase (DAO) are released from damaged enterocytes into the systemic circulation, leading to elevated serum DAO levels that correlate with the extent of epithelial barrier disruption (Yu et al., 2020). Compared with the Control group, serum DAO levels were significantly elevated in the model group (Figure 3A), indicating intestinal mucosal injury and increased gut permeability in the disease model. Treatment with Rifaximin, TXYF-L, and TXYF-H significantly reduced DAO levels compared with the IBS-D group, suggesting improved intestinal barrier function.

Furthermore, Rifaximin, TXYF-L, and TXYF-H treatment significantly decreased serum TNF-α levels while increasing those of IL-10 compared with the IBS-D group (Figure 3B). This reciprocal change in inflammatory cytokines was mirrored at the transcriptional level in colon tissue (Figure 3C), where TNF-α mRNA expression was downregulated, and IL-10 mRNA was upregulated. These results collectively demonstrate that TXYF effectively attenuated chronic low-grade inflammation in IBS-D rats.

### 3.4 TXYF modulated gut microbiota structure in IBS-D rats

**FIGURE 4:**
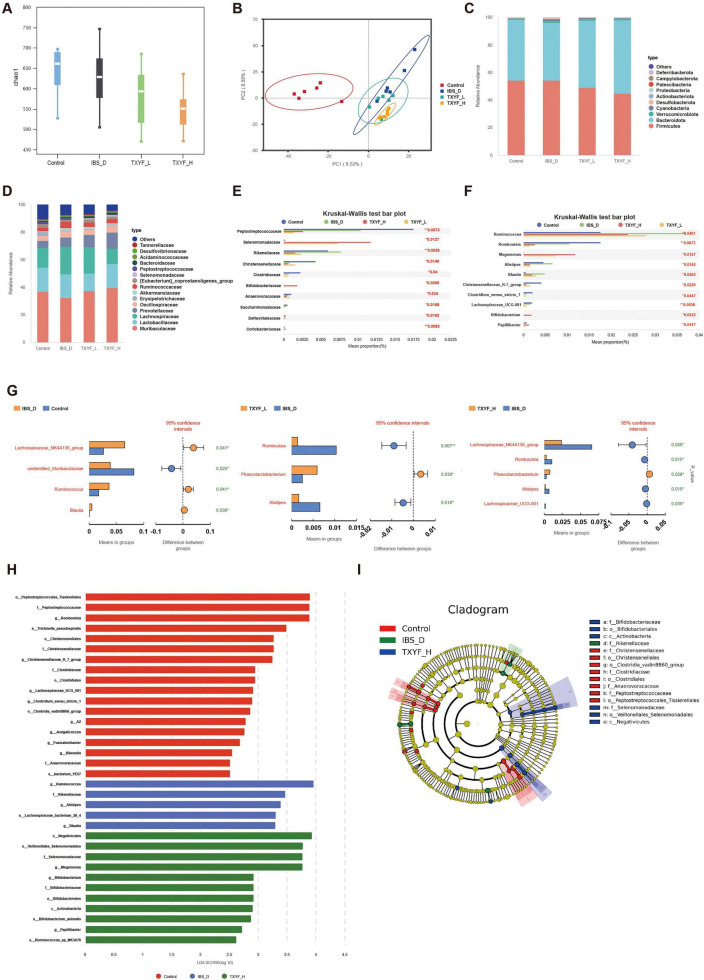
TXYF reshaped the gut microbiota composition in IBS-D rats (*n* = 6). **(A)** Chao1 index. **(B)** PCA score plots based on the ASVs level. **(C)** Bacterial community composition at the phylum level. **(D)** Bacterial community composition at the family level. **(E)** Kruskal-Wallis H test bar plot at the family level. **(F)** Kruskal-Wallis H test bar plot at the genus level. **(G)** *T*-test for different groups. **(H)** LDA distribution. **(I)** Cladogram generated by LEfSe. *p < 0:05, **p < 0:01.

Our initial investigation focused on analyzing shifts in the intestinal microbial community structure in rats. Rarefaction curves plateaued in all groups, indicating sufficient sequencing depth for subsequent analyses (Supplementary Figure S1A). Based on this, we evaluated the abundance and diversity of the community using the Chao1, Shannon, and Simpson indices (Figure 4A and Supplementary Figures S1B,C). The IBS-D group exhibited a decreasing trend in the Chao1 index compared with the Control group. Notably, relative to the IBS-D group, TXYF-L and TXYF-H treatments further reduced the Chao1 values (indicating decreased species richness) while increasing Shannon and Simpson indices (suggesting improved species diversity). However, these inter-group differences did not achieve statistical significance, potentially due to substantial intra-group biological variability and limited sample size. Principal component analysis (PCA) of β-diversity revealed distinct clustering among the Control, IBS-D, and TXYF-H groups (Figure 4B), suggesting that TXYF-H treatment substantially altered the gut microbial composition in IBS-D rats.

To comprehensively assess TXYF-induced microbial community alterations, we further performed taxonomic profiling at both the phylum and family levels (Figures 4C,D). We conducted intergroup Kruskal-Wallis tests at the phylum level; a significant difference was observed in the abundance of *Patescibacteria*, which accounted for approximately 0.0005% of the community (Supplementary Figure S2). At the family level (Figure 4E), the top 10 taxa showing the most significant changes in relative abundance were: *Peptostreptococcaceae*, *Selenomonadaceae*, *Rikenellaceae*, *Christensenellaceae*, *Clostridiaceae*, *Bifidobacteriaceae*, *Anaerovoracaceae*, *Saccharimonadaceae*, *Defluviitaleaceae*, and *Coriobacteriaceae*. At the genus level (Figure 4F), significant intergroup variation was observed in *Ruminococcus, Romboutsia, Megamonas, Alistipes, Blautia, Christensenellaceae_R-7_group, Clostridium_sensu_stricto_1, Lachnospiraceae_UCG-001, Bifidobacterium*, and *Papillibacter* in different groups. Subsequently, taxa showing statistically significant differences at the genus level were identified through *t*-tests (Figure 4G). *Lachnospiraceae NK4A136_group, unidentified Muribaculaceae, Ruminococcus*, and *Blautia* differed significantly between the IBS-D group and the Control group. *Romboutsia, Phascolarctobacterium*, and *Alistipes* showed significant differences between the TXYF-L group and the IBS-D group. Meanwhile, *Lachnospiraceae_NK4A136_group, Romboutsia, Phascolarctobacterium, Alistipes*, and *Lachnospiraceae_UCG-001* were significantly altered in the TXYF-H group compared with the IBS-D group.

Linear discriminant analysis effect size (LEfSe) was used to identify group-specific microbial biomarkers, applying a multi-level species discrimination approach with an LDA score threshold of 2.5 (Figures 4H,I). The top five microbial biomarkers were as follows: in the Control group, *o_Peptostreptococcales_Tissierellales, f_Peptostreptococcaceae, g_ Romboutsia, s_Trichinella_pseudospiralis*, and *o_Christensenellales*; in the IBS-D group, *g_Ruminococcus, f_Rikenellaceae, g_Alistipes, s_Lachnospiraceae_bacterium_28_4*, and *g_Blautia*; and in the TXYF-H group, *c_Negativicutes*, *o_Veillonellales_Selenomonadales*, *f_Selenomonadaceae*, *g_Megamonas*, and *g_Bifidobacterium*. These findings indicate that TXYF restores gut microbiota homeostasis in IBS-D, suggesting a potential therapeutic mechanism for this condition.

### 3.5 TXYF reshaped TRP-related metabolite profiles in the intestinal contents of IBS-D rats

**FIGURE 5:**
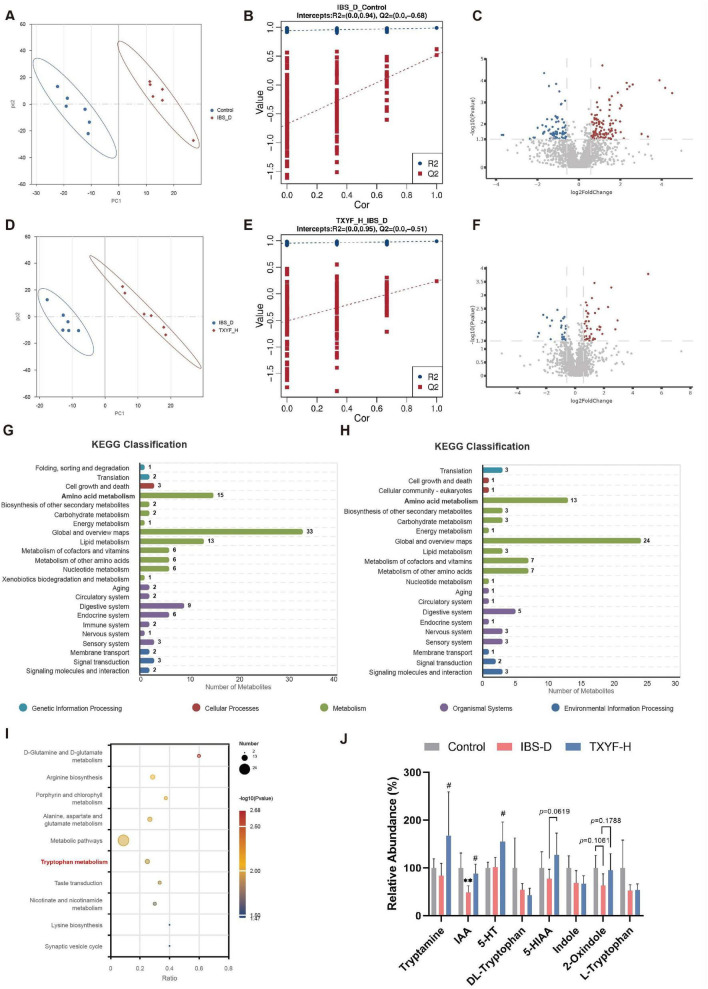
TXYF restored the metabolic disorders under IBS-D and enriched microbiota-derived TRP metabolites (*n* = 6). **(A)** PLS-DA score plot for the Control and IBS-D groups. **(B)** Permutation test of the PLS-DA model. **(C)** Volcano plot of the IBS-D group vs. the Control group. **(D)** PLS-DA score plot for the IBS-D and the TXYF-H groups. **(E)** Permutation test of the PLS-DA model. **(F)** Volcano plot of the TXYF-H group vs. the IBS-D group. **(G)** KEGG classification of the IBS-D group vs. the Control group. **(H)** KEGG classification of the IBS-D group vs. the TXYF-H group. **(I)** Enriched KEGG pathways in the TXYF-H group vs. the IBS-D group. **(J)** Relative abundance of microbiota-derived TRP metabolites in colonic contents. Compared with the Control group, **p < 0.01; compared with the IBS-D group, #*p* < 0.05.

Alterations in gut microbiota composition induce functional remodeling of metabolic pathways, where microbiota-derived metabolites serve as crucial signaling molecules in bidirectional host-microbe communication. Therefore, we used LC-MS technology to detect the metabolome of intestinal contents. The partial least squares-discriminant analysis (PLS-DA) model, constructed using combined positive and negative ion data, revealed significant metabolic separation between the Control and IBS-D groups, as well as between the TXYF-H and IBS-D groups (Figures 5A,D). The permutation test results indicated that the models were not overfitted, supporting their validity and reliability (Figures 5B,E). These results emphasize that the intestinal content metabolic profile of IBS-D rats was altered compared with the Control group, whereas TXYF-H treatment reshaped this profile.

Next, we screened the metabolites that were significantly altered after IBS-D induction and TXYF-H treatment based on the criteria of *p* ≤ 0.05, |log_2_FoldChange| ≥ 0.58, and VIP ≥ 1 (Supplementary Table S1). Compared with the Control group, the IBS-D group showed 184 significantly altered metabolites (112 upregulated, 72 downregulated; Figure 5C). The TXYF-H group exhibited 74 significantly changed metabolites compared with the IBS-D group (48 upregulated, 26 downregulated; Figure 5F). According to the KEGG classification, the majority of these metabolites were categorized into pathways related to amino acid metabolism (Figures 5G,H). KEGG pathway analysis revealed significant enrichment of the TRP metabolic pathway in the TXYF-H group compared with the IBS-D group (Figure 5I). As shown in Figure 5J, compared with the Control group, IBS-D rats exhibited decreased levels of L-TRP, 5-hydroxyindole-3-acetic acid (5-HIAA), and 2-oxindole with a particularly significant reduction in indole-3-acetic acid (IAA) levels. TXYF treatment increased the levels of tryptamine, IAA, and 5-hydroxytryptamine (5-HT). 5-HIAA and 2-oxindole levels in the TXYF-H group were elevated but not significantly different from those in the IBS-D group. Collectively, these findings indicate that modulation of the TRP metabolite profile may contribute to the therapeutic effects of TXYF.

### 3.6 TXYF promoted intestinal barrier integrity and activated the AhR pathway in IBS-D rats

**FIGURE 6:**
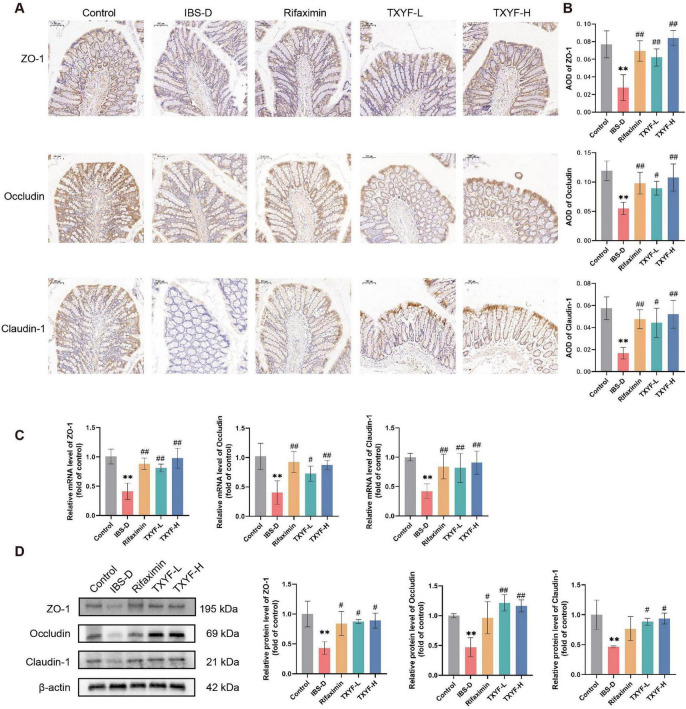
TXYF attenuated the loss of intestinal TJ proteins in IBS-D rats. **(A)** Representative immunohistochemical images of ZO-1, Occludin, and Claudin-1 in the colon (200 × ). **(B)** Quantification of ZO-1, Occludin, and Claudin-1 AOD in the colon (*n* = 5). **(C)** Relative mRNA expression levels of ZO-1, Occludin, and Claudin-1 in the colon (*n* = 6). **(D)** Relative protein expression levels of ZO-1, Occludin, and Claudin-1 in the colon (*n* = 3). Values are expressed as the mean ± SD. Compared with the Control group, **p < 0.01; compared with the IBS-D group, #*p* < 0.05, ##*p* < 0.01.

Intestinal barrier integrity is closely linked to gut microbiota composition. A compromised intestinal barrier permits bacterial translocation and pathogen invasion, thereby triggering immune responses (Shimbori et al., 2022). TJ proteins are specialized connections between epithelial cells that function primarily to seal intercellular spaces and prevent the uncontrolled passage of substances through these spaces, serving as a “gate and fence” to maintain the selective permeability of the cell layer (Zihni et al., 2016). The IHC experiments revealed significantly decreased expression of ZO-1, Occludin, and Claudin-1 in the colon of IBS-D rats compared with controls, while Rifaximin and TXYF treatments reversed these reductions (Figures 6A,B). Consistent with these findings, both RT-qPCR and Western blot analyses demonstrated downregulation of these TJs at both transcriptional and translational levels in the IBS-D group (Figures 6C,D). Conversely, the mRNA and protein expression of TJs was significantly upregulated in the Rifaximin, TXYF-L, and TXYF-H groups. Together, these results suggest TXYF ameliorated intestinal barrier dysfunction by enhancing TJ protein expression.

**FIGURE 7:**
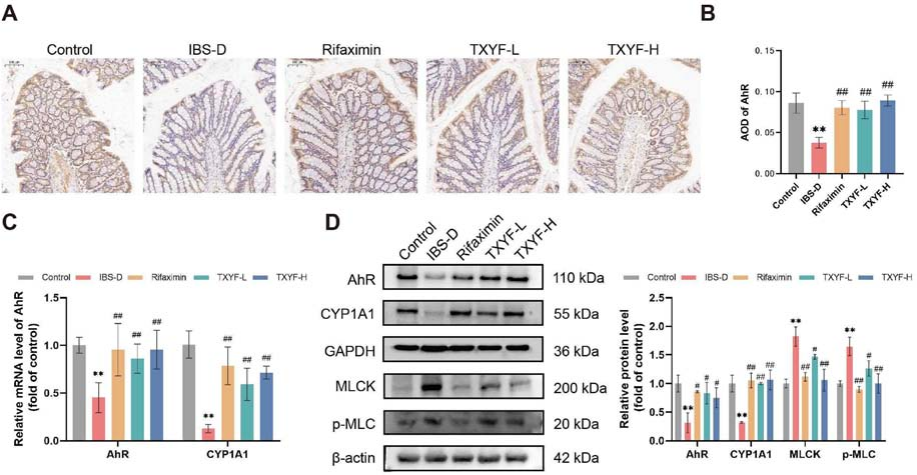
TXYF activated the AhR pathway in IBS-D rats. **(A)** Representative immunohistochemical images of AhR in the colon (200 × ). **(B)** Quantification of AhR AOD in the colon (*n* = 5). **(C)** Relative mRNA expression levels of AhR and CYP1A1 in the colon (*n* = 6). **(D)** Relative protein expression levels of AhR, CYP1A1, MLCK, and p-MLC in the colon (*n* = 3). Values are expressed as the mean ± SD. Compared with the Control group, **p < 0.01; compared with the IBS-D group, #*p* < 0.05, ##*p* < 0.01.

Based on our untargeted metabolomics analysis, we observed alterations in TRP metabolites, including 5-HIAA, IAA, tryptamine, and 2-oxindole, in the intestinal contents. Recent studies have shown that TRP metabolites activate the AhR, thereby upregulating TJ protein expression and restoring intestinal barrier function (Wang et al., 2024). We therefore investigated whether TXYF exerts protective effects on the intestinal barrier in IBS-D rats via the AhR pathway. As expected, AhR expression in the intestinal mucosa was decreased in the IBS-D group, whereas treatment with Rifaximin, TXYF-L, and TXYF-H restored its levels (Figures 7A,B). Consistent with these observations, both RT-qPCR and Western blot analyses revealed significant downregulation of AhR and its downstream target CYP1A1 in the IBS-D group relative to the Control group. Notably, TXYF treatment effectively restored their expression levels (Figures 7C,D), indicating activation of the AhR signaling pathway. Further analysis of the AhR downstream MLCK/p-MLC pathway revealed significantly elevated expression of both MLCK and p-MLC proteins in the IBS-D group (Figure 7C). TXYF treatment effectively reversed these changes, suggesting its modulatory effect on the MLCK/p-MLC signaling axis. Taken together, the activation of AhR by TXYF is identified as a key mechanism underlying its protective effect on the intestinal barrier.

### 3.7 FMT from TXYF-treated donors recapitulated the therapeutic effects of TXYF in IBS-D rat models

**FIGURE 8:**
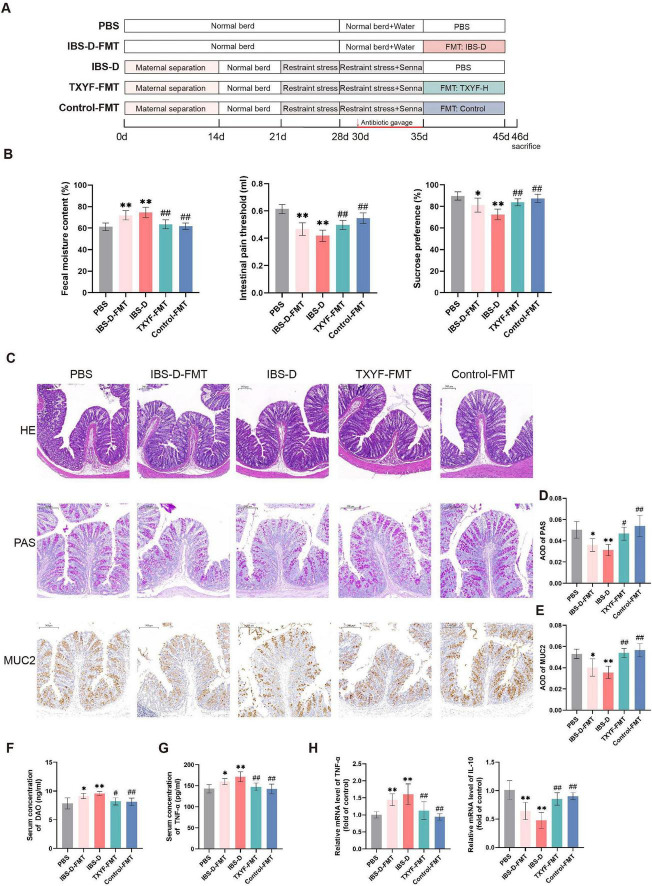
FMT of TXYF-shaped microbiota recapitulated the therapeutic effect of TXYF in IBS-D rats. **(A)** Schematic of the FMT procedure in rats. **(B)** Fecal moisture content (*n* = 8), intestinal pain threshold (*n* = 8), and sucrose preference (*n* = 6) in each group. **(C)** Representative images of HE staining (100 × ), PAS staining (150 × ), and MUC2 immunohistochemistry (150 × ) in the colon. **(D,E)** Quantification of PAS, MUC2 AOD in the colon (*n* = 5). **(F)** Serum concentration of DAO (*n* = 6). **(G)** Serum concentration of TNF-α (*n* = 6). **(H)** Relative mRNA expression levels of TNF-α, IL-10 (*n* = 6). Values are expressed as the mean ± SD. Compared with the PBS group, *p < 0.05, **p < 0.01; compared with the IBS-D group, #*p* < 0.05, ##*p* < 0.01.

Fecal microbiota from IBS-D rats was transplanted into pseudo-germ-free rats to establish a causal link between dysbiosis and intestinal barrier damage. For the FMT treatment group, we selected the TXYF-H donor microbiota based on its superior efficacy in modulating microbial communities compared with the TXYF-L group. Fecal microbiota from the Control group was transplanted in parallel as a positive control (Figure 8A). Compared with the PBS group, both the IBS-D-FMT and IBS-D groups displayed significantly increased FMC, a lowered visceral pain threshold, and reduced sucrose preference (Figure 8B). Importantly, the TXYF-FMT group showed marked improvements in these parameters compared with the IBS-D group (Figure 8B).

HE staining revealed no histopathological abnormalities in the colon of any group (Figure 8C). Both PAS staining and IHC demonstrated reduced goblet cell numbers and MUC2 protein expression in the IBS-D-FMT group compared with the PBS group (Figures 8C–E). Of note, the TXYF-FMT and Control-FMT groups exhibited restored goblet cell populations and enhanced MUC2 expression relative to the IBS-D group.

To assess the impact of FMT on intestinal barrier function and inflammation, we measured serum DAO and cytokine levels. Barrier disruption, indicated by elevated serum DAO, was observed in the IBS-D-FMT and IBS-D groups compared with the PBS group, whereas TXYF-FMT and Control-FMT reduced DAO to lower levels (Figure 8F). Similarly, serum TNF-α levels were significantly increased in the IBS-D-FMT and IBS-D groups, while both the TXYF-FMT and Control-FMT groups exhibited significantly lower TNF-α levels relative to the IBS-D group (Figure 8G). Simultaneously, TNF-α mRNA expression in the colon was markedly upregulated, whereas IL-10 mRNA was downregulated in the IBS-D-FMT and IBS-D groups (Figure 8H). Importantly, the FMT from TXYF-treated donors effectively reversed these pro-inflammatory changes. Together, these findings suggest that gut microbiota dysbiosis contributed to the development of IBS-D and that the therapeutic benefits of TXYF were mediated, at least in part, through modulation of the gut microbiota.

### 3.8 FMT from TXYF-treated donors enhanced intestinal epithelial barrier function via AhR activation in IBS-D models

**FIGURE 9:**
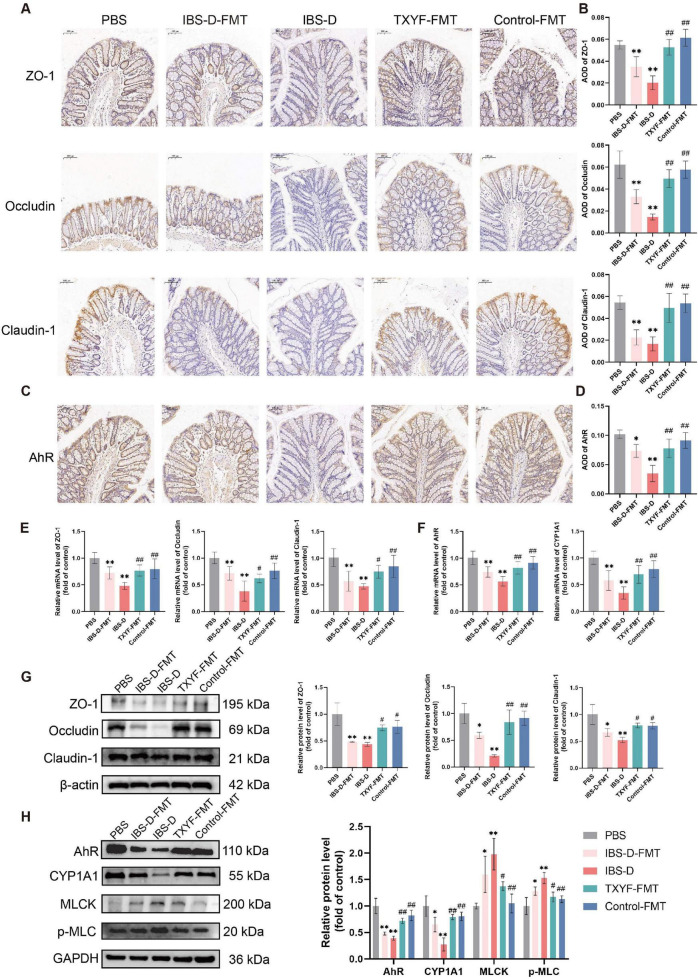
FMT affected the intestinal barrier and AhR pathway in IBS-D rats. **(A)** Representative immunohistochemical images of ZO-1, Occludin, and Claudin-1 in the colon (200 × ). **(B)** Quantification of ZO-1, Occludin, and Claudin-1 in the colon (*n* = 5). **(C)** Representative immunohistochemical images of AhR in the colon (200 × ). **(D)** Quantification of AhR in the colon (*n* = 5). **(E)** Relative mRNA expression levels of ZO-1, Occludin, and Claudin-1 in the colon (*n* = 6). **(F)** Relative mRNA expression levels of AhR and CYP1A1 in the colon (*n* = 6). **(G)** Relative protein expression levels of ZO-1, Occludin, and Claudin-1 in the colon (*n* = 3). **(H)** Relative protein expression levels of AhR, CYP1A1, MLCK, and p-MLC in the colon (*n* = 3). Values are expressed as the mean ± SD. Compared with the PBS group, *p < 0.05, **p < 0.01; compared with the IBS-D group, #*p* < 0.05, ##*p* < 0.01.

Transplantation of IBS-D microbiota impaired barrier integrity, as evidenced by reduced expression of the TJ proteins ZO-1, Occludin, and Claudin-1 (Figures 9A,B). Relative to the PBS group, the IBS-D-FMT and IBS-D groups exhibited downregulation of AhR and CYP1A1 at both the gene and protein levels, along with activation of the MLCK/p-MLC pathway (Figures 9C–H). In contrast, the TXYF-FMT group showed restored TJ protein expression, upregulation of AhR and CYP1A1, and inhibition of the MLCK/p-MLC pathway (Figures 9A–H). Collectively, these findings demonstrate that intestinal barrier function is critically regulated by the gut microbiota and that TXYF restores TJ integrity in IBS-D rats by modulating microbiota and activating the AhR pathway.

## 4 Discussion

Current research suggests that microbiota-derived metabolites, especially from TRP, regulate intestinal barrier function and likely contribute to IBS-D progression (Ghosh et al., 2021; Li et al., 2024; Wang L. et al., 2025). TXYF is a classic herbal formula extensively studied for the management of IBS-D (Long et al., 2025; Lu and Zhang, 2023). In this study, we demonstrate that TXYF restores intestinal barrier integrity by increasing the abundance of probiotic *Bifidobacterium* and key TRP metabolites (IAA, 5-HIAA, tryptamine, 2-oxindole), thereby activating the AhR pathway and inhibiting the MLCK/p-MLC signaling axis. Moreover, the FMT experiment showed that microbiota from TXYF-treated rats conferred these therapeutic benefits, suggesting TXYF represents a viable strategy for targeting gut microbiota in IBS-D.

The IBS-D rat model with liver depression-spleen deficiency syndrome was established using a combination of maternal separation, restraint stress, and *Sennae folium* decoction gavage—an approach widely recognized for inducing IBS-D phenotypes in preclinical studies (Wei et al., 2024; Zhang S. et al., 2024). Rifaximin, recommended for IBS-D treatment (Lacy et al., 2021) and reported to alleviate gut dysbiosis and elevate levels of TRP-related metabolites (Zhang Y. et al., 2024), was selected as the positive control. After TXYF treatment, the symptoms of diarrhea and abdominal pain, as well as the levels of inflammatory factors, were significantly ameliorated (Ng et al., 2018; Aguilera-Lizarraga et al., 2022). Furthermore, TXYF effectively restored goblet cell numbers and mucin secretion, while also markedly reducing serum DAO levels, indicating substantial improvement in intestinal barrier function. Together, these effects contribute to the restoration of intestinal homeostasis and amelioration of IBS-D symptoms.

The human gut microbiota, comprising approximately 10^14^ microbial cells (Backhed et al., 2005), engages in a multifaceted symbiotic relationship (Kayama et al., 2020) and modulates local and systemic physiology (LeBlanc et al., 2013). The literature presents a complex picture of microbiota alterations in IBS. Although reduced α-diversity and an elevated *Firmicutes/Bacteroidetes* (F/B) ratio are frequently reported in IBS (Duan et al., 2019), some studies have found no significant difference in these metrics in IBS-D patients versus healthy controls (Tap et al., 2017). This lack of consensus regarding the IBS-D microbiota signature may be explained by inter-study variations in human populations, geographic regions, and dietary habits (Shabani et al., 2025). A similar inconsistency exists in IBS-D animal models (Chen et al., 2025; Hang et al., 2022). Consistent with this, our study observed only modest intergroup differences in the F/B ratio and α-diversity at the phylum level. Despite these subtle shifts at the phylum level, deeper analysis revealed significant gut microbiota disruption in IBS-D rats, particularly at finer taxonomic resolutions. At the genus level, we observed a marked increase in the relative abundance of pro-inflammatory opportunistic pathogens, including *Ruminococcus, Lachnospiraceae NK4A136_group*, and *Blautia*, which have been linked to intestinal mucin degradation, inflammation, and anxiety-like behaviors (Crost et al., 2016; Liu D. et al., 2023; Bolte et al., 2021). Conversely, we noted a decrease in mucin-promoting *Muribaculaceae*, a group that may support beneficial bacteria such as *Bifidobacterium* and *Lactobacillus* through cross-feeding interactions (Zhu et al., 2024). TXYF treatment reversed these changes in microbiota. Additionally, TXYF treatment significantly reduced the relative abundance of *Romboutsia* and *Alistipes*, while increasing the proportions of *Megamonas*, *Bifidobacterium*, and *Phascolarctobacterium*. These microbial shifts may contribute to the dysregulation of lipid metabolism, TRP metabolism, and reduced production of short-chain fatty acids (SCFAs) (Liu J. et al., 2023; Wu et al., 2024; Yin et al., 2023; Ehrlich et al., 2020). Significantly, LEfSe analysis further identified *Bifidobacterium* as a high-scoring beneficial microbe, implicating it in the mediation of TXYF’s protective effect against IBS-D (Turroni et al., 2011). Although we did not directly investigate *Bifidobacterium* in our IBS-D model, previous literature reports that *Bifidobacterium* supplementation can reduce gastrointestinal symptom severity and improve psychological wellbeing in IBS-D patients (Lewis et al., 2020). Notably, in rat models of IBS, *B. longum* relieves symptoms by enhancing mucosal repair and lysozyme production, possibly via upregulation of Paneth cell-derived WNT3A and TGF-β (Zhou et al., 2020). These findings support *Bifidobacterium* as a therapeutic target of TXYF in the treatment of IBS-D.

Converging evidence from clinical and preclinical studies implicates disrupted TRP metabolism in the gastrointestinal symptoms of IBS-D, highlighting the involvement of the gut microbiota axis (Mujagic et al., 2022; Han et al., 2022; Ouyang et al., 2024). In this context, therapeutic strategies aimed at restoring TRP metabolic homeostasis in the dysbiotic microbiota hold significant promise for IBS-D. We observed marked alterations in TRP metabolism in IBS-D rats. TXYF treatment modulated intestinal content metabolism and increased the levels of specific TRP-derived metabolites, including IAA, 5-HIAA, 5-HT, tryptamine, and 2-oxindole. Notably, elevated IAA and 5-HIAA—potentially driven by *Bifidobacterium*—have been implicated in intestinal barrier restoration (Laursen et al., 2021; Han et al., 2023; Min et al., 2024). Consistent with this, *Bifidobacterium* promotes TRP conversion to 5-HT, thereby alleviating anxiety- and depression-like behaviors (Pan et al., 2025; Wang J. et al., 2025). Meanwhile, *Clostridium sporogenes* generates tryptamine through the decarboxylation of TRP (Williams et al., 2014). Additionally, studies have reported that the microbiota is necessary for the production of 2-oxindole (Dong et al., 2020). Collectively, these findings indicate that TXYF restores microbiota-associated TRP metabolism, representing a key mechanism for its therapeutic efficacy.

TRP metabolites—such as IAA, 5-HIAA, tryptamine, and 2-oxindole—are endogenous ligands of AhR, and activation of the AhR signaling pathway plays a critical role in maintaining intestinal barrier integrity (Wang L. et al., 2025; Meynier et al., 2022). Upon binding, AhR translocates to the nucleus (Li et al., 2024; Dong et al., 2020) and binds to xenobiotic response elements (XREs) to drive the expression of TJ proteins (Ganapathy et al., 2023). TXYF treatment elevated the expression levels of TJ proteins (including ZO-1, Occludin, and Claudin-1), thereby enhancing intestinal barrier function in IBS-D rats. Concurrently, TXYF treatment upregulated AhR and CYP1A1, indicating activation of the AhR pathway. In addition to directly promoting the transcription of TJ protein genes, AhR can also inhibit the MLCK/p-MLC pathway to maintain TJ integrity and ameliorate intestinal epithelial barrier damage (Han et al., 2016). Increased epithelial MLC phosphorylation, which correlates with elevated intestinal permeability, has been observed in IBS-D patients (Wu et al., 2017). In IBS-D rats, protein levels of MLCK and p-MLC were elevated, and this increase was significantly attenuated by TXYF treatment. Given the established link between IBS-D and gut dysbiosis, FMT has garnered interest as a therapeutic strategy (De Palma et al., 2017). Our FMT experiment showed that microbiota from IBS-D donors transferred IBS-D-like symptomatology and impaired intestinal barrier function to recipient rats, while microbiota from TXYF-treated donors alleviated these symptoms and activated the AhR pathway. Collectively, our data demonstrate that TXYF improves intestinal TJ integrity in IBS-D by activating the AhR and inhibiting the MLCK/p-MLC pathway, an effect mediated, at least in part, through gut microbiota modulation.

Although our study provides novel insights into the pathogenesis of IBS-D and the therapeutic mechanism of TXYF, several limitations should be acknowledged. First, the rat model used in our study, established using a widely adopted triple-factor approach, cannot fully recapitulate the complex etiology, pathophysiology, and clinical manifestations of human IBS-D. Second, because pregnant rats were used, both male and female offspring were included. It is worth noting that sex differences have been reported to affect IBS-D susceptibility, as well as gut microbiota and metabolism, potentially introducing bias (Francis-Malave et al., 2023; Pigrau et al., 2016). To minimize potential bias arising from sex differences, we maintained a balanced male-to-female ratio across all groups. Nevertheless, our study may still be subject to a certain degree of bias. Third, we did not determine whether the chemical constituents of TXYF can directly activate the AhR pathway. Finally, a definitive causal link between specific bacterial species (or their associated metabolites) and intestinal barrier function could not be established in our study. These limitations highlight important directions for future research. Notably, the precise microbiota-metabolite-AhR network in patients requires clinical validation, whereas animal studies with expanded sample sizes are necessary to assess sex-based differences in various indicators (Clayton and Collins, 2014). Additionally, *in vitro* studies are needed to establish direct causality between barrier function and specific candidates such as *Bifidobacterium* and TRP metabolites. Ultimately, studies employing intestinal epithelium-specific AhR knockout mice are warranted to define the functional role of AhR in IBS-D pathology.

## 5 Conclusion

TXYF ameliorated low-grade colonic inflammation and intestinal barrier dysfunction by restoring gut microbiota in an IBS-D model. These effects may be mediated through the activation of AhR by microbiota-derived TRP metabolites, with AhR serving as a key target of TXYF. Together, this study provides novel mechanistic insights and identifies promising therapeutic targets for IBS-D.

## Data Availability

The datasets presented in this study can be found in online repositories. The names of the repository/repositories and accession number(s) can be found at: https://www.ncbi.nlm.nih.gov/, PRJNA1437742.

## Footnotes

1
https://magic-plus.novogene.com/
