# Supplementary material for: Tongxie Yaofang ameliorates IBS-D by targeting the gut microbiota-derived tryptophan metabolites and AhR signaling axis
Source: Front Microbiol. 2026 Apr 30;17:1786701. doi: 10.3389/fmicb.2026.1786701 (PMC13173512; doi:10.3389/fmicb.2026.1786701)
Supplement: Supplementary file 1 [file Supplementary_file_1.docx]

**1.**


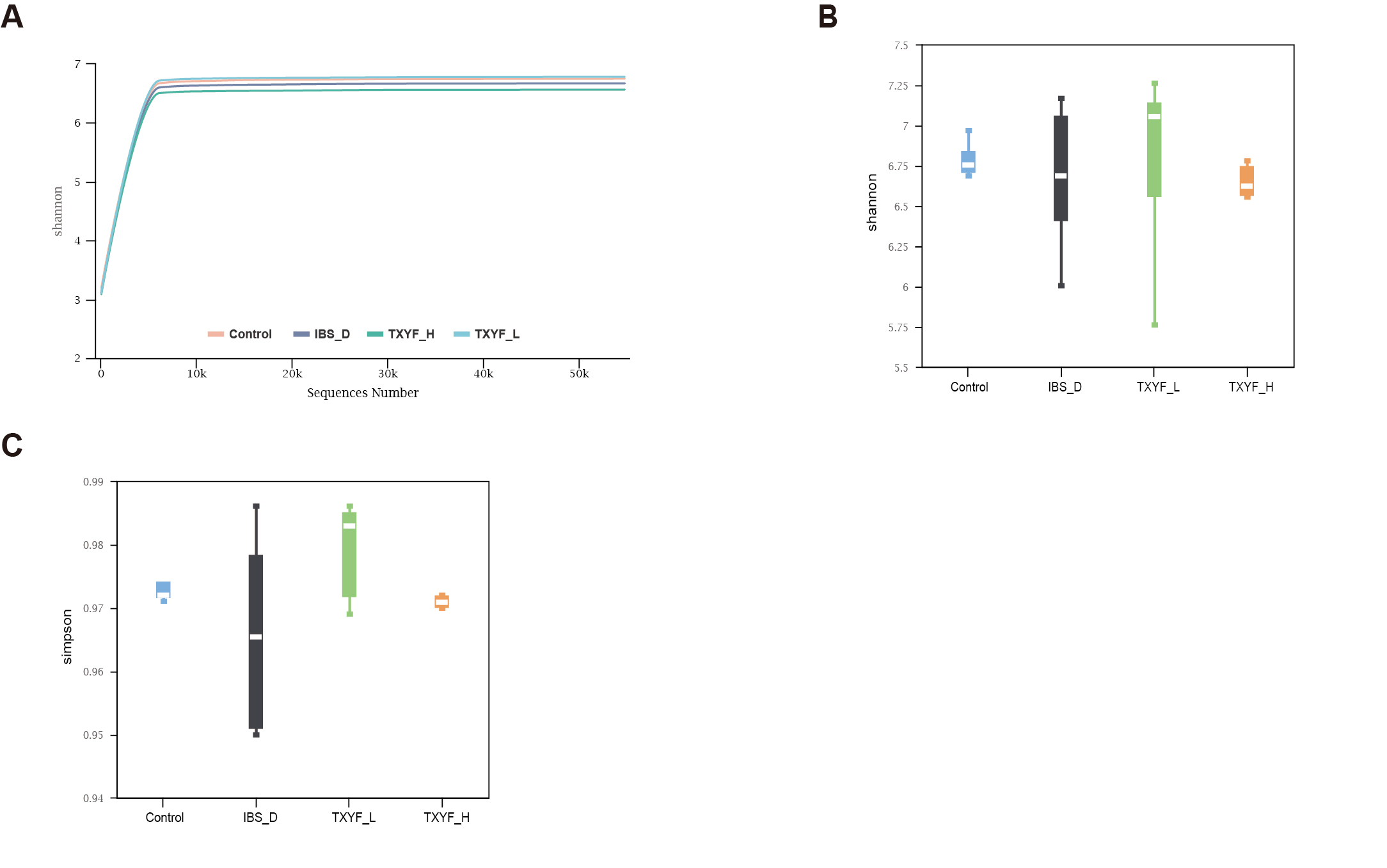


Fig. S1. TXYF modulated the structure and diversity of the gut microbiota (n=6). (A) Rarefaction curves. (B) Shannon indexes. (C) Simpson indexes.

2.


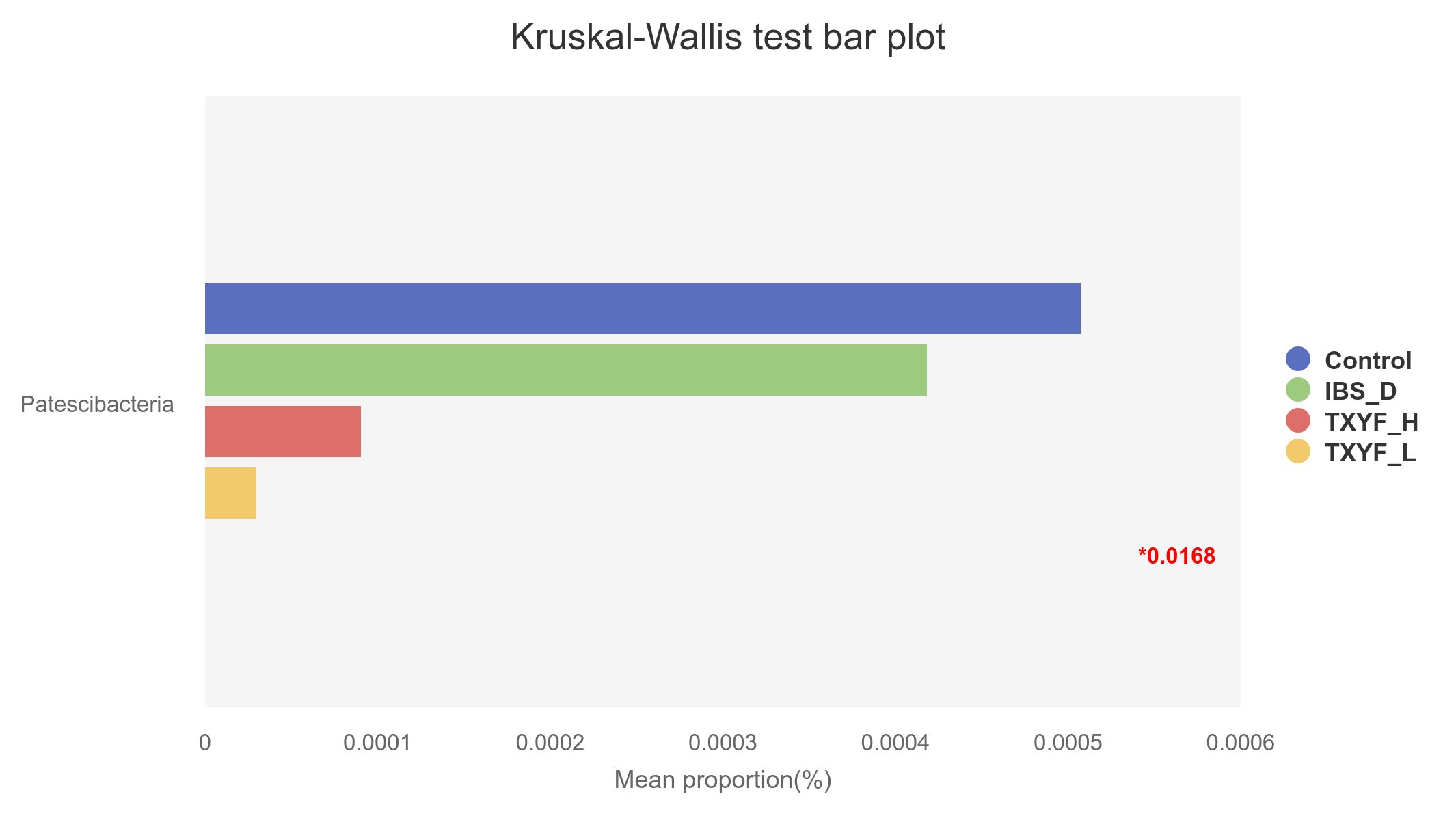


Fig. S2. Kruskal-Wallis H test bar plot at the phylum level.

3.

Table S1 The differential metabolites in each group

| Comparison group | Name | FC | log2FC | Pvalue | VIP | Up.Down |
| --- | --- | --- | --- | --- | --- | --- |
| IBS-D.vs.Control | QMK | 2.189315 | 1.130479 | 1.99591E-05 | 2.041486 | up |
| IBS-D.vs.Control | Saccharin | 0.312104 | -1.6799 | 4.52993E-05 | 2.546082 | down |
| IBS-D.vs.Control | Adenine | 14.83782 | 3.891207 | 9.6902E-05 | 2.458928 | up |
| IBS-D.vs.Control | Auramine | 4.93646 | 2.303477 | 0.000126952 | 2.111493 | up |
| IBS-D.vs.Control | LPE O-16:2 | 0.466822 | -1.09906 | 0.000141745 | 2.196736 | down |
| IBS-D.vs.Control | Michler's ketone | 6.041561 | 2.594921 | 0.000150889 | 2.123559 | up |
| IBS-D.vs.Control | 2-methyl-6-{[(5-phenyl-2-thienyl)carbonyl]amino}benzoic acid | 0.366493 | -1.44814 | 0.000176982 | 2.36369 | down |
| IBS-D.vs.Control | Mirtazapine-d3 | 5.445801 | 2.445144 | 0.000184556 | 2.127263 | up |
| IBS-D.vs.Control | LysoPC 12:1 | 1.981579 | 0.986651 | 0.000211296 | 2.177341 | up |
| IBS-D.vs.Control | 2'-Deoxyadenosine | 17.96507 | 4.167123 | 0.000215387 | 2.536733 | up |
| IBS-D.vs.Control | Estriol | 5.023006 | 2.328551 | 0.000245984 | 2.332856 | up |
| IBS-D.vs.Control | 2-(1H-indol-3-yl)acetic acid | 0.51873 | -0.94694 | 0.00031563 | 2.398626 | down |
| IBS-D.vs.Control | S-Adenosylmethionine | 3.67412 | 1.877399 | 0.000356074 | 1.975393 | up |
| IBS-D.vs.Control | 1-Methylguanosine | 22.6695 | 4.502681 | 0.000375694 | 2.504553 | up |
| IBS-D.vs.Control | Biliverdin | 2.998755 | 1.584364 | 0.000526302 | 2.092182 | up |
| IBS-D.vs.Control | N-Acetyl-aspartic acid | 2.762996 | 1.466233 | 0.000730272 | 2.206208 | up |
| IBS-D.vs.Control | 3-morpholino-5,6-diphenylpyridazine-4-carbonitrile | 2.770818 | 1.470312 | 0.000806019 | 2.026357 | up |
| IBS-D.vs.Control | mesaconic acid | 0.628944 | -0.669 | 0.000845425 | 2.009291 | down |
| IBS-D.vs.Control | Gibberellic acid | 1.824815 | 0.86775 | 0.001007672 | 1.727503 | up |
| IBS-D.vs.Control | Ergosterol peroxide | 0.557609 | -0.84267 | 0.001163436 | 1.703231 | down |
| IBS-D.vs.Control | 4-Hydroxy-3-methylbenzoic acid | 0.546994 | -0.8704 | 0.001255195 | 2.057827 | down |
| IBS-D.vs.Control | Sulfaquinoxaline | 2.832157 | 1.501901 | 0.001312456 | 2.077215 | up |
| IBS-D.vs.Control | Hydrocortisone acetate | 4.08302 | 2.029637 | 0.001318817 | 1.97806 | up |
| IBS-D.vs.Control | N6-Succinyl Adenosine | 4.177658 | 2.062694 | 0.001492005 | 1.886841 | up |
| IBS-D.vs.Control | FNK | 1.867284 | 0.900941 | 0.00166925 | 2.262479 | up |
| IBS-D.vs.Control | Morphine | 3.944232 | 1.979744 | 0.001920019 | 2.111273 | up |
| IBS-D.vs.Control | RLK | 2.785284 | 1.477824 | 0.002258355 | 1.97553 | up |
| IBS-D.vs.Control | 16,16-Dimethyl prostaglandin A2 | 2.04961 | 1.035349 | 0.002317942 | 1.768434 | up |
| IBS-D.vs.Control | 2-oxa-4-azatetracyclo[6.3.1.1~6,10~.0~1,5~]tridecan-3-one | 1.529229 | 0.612804 | 0.003584618 | 2.108273 | up |
| IBS-D.vs.Control | Benzoic acid | 1.824248 | 0.867302 | 0.00375804 | 1.856195 | up |
| IBS-D.vs.Control | Deoxycholic acid | 1.724196 | 0.785923 | 0.00396338 | 2.154766 | up |
| IBS-D.vs.Control | Dl-3-Hydroxynorvaline | 2.753774 | 1.46141 | 0.004247007 | 1.879483 | up |
| IBS-D.vs.Control | Cholest-4-en-3-one | 0.455007 | -1.13604 | 0.00434218 | 2.141983 | down |
| IBS-D.vs.Control | 2-[5-(2-hydroxypropyl)oxolan-2-yl]propanoic acid | 0.470194 | -1.08867 | 0.004767829 | 1.910619 | down |
| IBS-D.vs.Control | Indole-3-acetic acid | 0.490649 | -1.02724 | 0.004904424 | 2.095535 | down |
| IBS-D.vs.Control | GPK | 1.676501 | 0.745453 | 0.005285142 | 1.776488 | up |
| IBS-D.vs.Control | Tretinoin | 0.484159 | -1.04645 | 0.00541721 | 1.109305 | down |
| IBS-D.vs.Control | 4-methoxy-6-[2-(4-methoxyphenyl)ethyl]-2H-pyran-2-one | 1.786128 | 0.836836 | 0.005658896 | 1.868241 | up |
| IBS-D.vs.Control | Cholecalciferol | 0.344843 | -1.53599 | 0.006004078 | 2.037226 | down |
| IBS-D.vs.Control | Azetidine-2-carboxylic acid | 0.573021 | -0.80334 | 0.006056244 | 1.884103 | down |
| IBS-D.vs.Control | Lovastatin | 1.679352 | 0.747904 | 0.006085724 | 1.883145 | up |
| IBS-D.vs.Control | gamma-Glutamylcysteine | 0.40275 | -1.31204 | 0.006172212 | 2.193314 | down |
| IBS-D.vs.Control | (S)-Equol | 1.884599 | 0.914257 | 0.006288101 | 1.78106 | up |
| IBS-D.vs.Control | (+/-)-Equol | 1.798592 | 0.846868 | 0.006293348 | 1.669214 | up |
| IBS-D.vs.Control | bicyclo[2.2.2]oct-2-en-1-yl 4-methylbenzene-1-sulfonate | 2.959626 | 1.565415 | 0.006696398 | 1.945296 | up |
| IBS-D.vs.Control | (3R)-8-hydroxy-3-(4-methoxyphenyl)-3,4-dihydro-1H-2-benzopyran-1-one | 3.419502 | 1.773786 | 0.006797124 | 1.778836 | up |
| IBS-D.vs.Control | VLK | 2.274702 | 1.185678 | 0.007241949 | 1.847483 | up |
| IBS-D.vs.Control | 3,8,9-trihydroxy-10-propyl-3,4,5,8,9,10-hexahydro-2H-oxecin-2-one | 0.502949 | -0.99152 | 0.007695027 | 1.904748 | down |
| IBS-D.vs.Control | 3-[(4-chlorophenyl)thio]-1-(3-pyridylmethyl)pyrrolidine-2,5-dione | 3.96475 | 1.98723 | 0.007746611 | 1.783154 | up |
| IBS-D.vs.Control | Glu-Val-Phe | 1.990226 | 0.992932 | 0.007797543 | 1.675907 | up |
| IBS-D.vs.Control | ELK | 1.943287 | 0.958499 | 0.00784632 | 1.511802 | up |
| IBS-D.vs.Control | LPS 14:0 | 0.465868 | -1.10201 | 0.008124684 | 1.312701 | down |
| IBS-D.vs.Control | 3-N-Methyl-L-histidine | 1.518703 | 0.602839 | 0.008140422 | 1.992096 | up |
| IBS-D.vs.Control | D-Ala-D-Ala | 1.572441 | 0.653006 | 0.00825813 | 1.888724 | up |
| IBS-D.vs.Control | 2-[(3S)-1-(2-Fluorobenzyl)-3-pyrrolidinyl]-1,3-benzothiazole | 0.605536 | -0.72371 | 0.00850744 | 1.873895 | down |
| IBS-D.vs.Control | Adenosine | 2.140982 | 1.098273 | 0.008825752 | 1.79857 | up |
| IBS-D.vs.Control | ethyl 4-({[(3-morpholinopropyl)amino]carbothioyl}amino)benzoate | 1.802702 | 0.850161 | 0.008921694 | 1.989646 | up |
| IBS-D.vs.Control | 19(R)-hydroxy Prostaglandin A2 | 1.971303 | 0.979149 | 0.008966251 | 1.443615 | up |
| IBS-D.vs.Control | N1-(6-methyl-4-oxo-3,4-dihydroquinazolin-2-yl)-4-nitrobenzamide | 1.684419 | 0.752251 | 0.009344953 | 1.695528 | up |
| IBS-D.vs.Control | Xanthohumol | 3.134535 | 1.648251 | 0.009358973 | 1.653354 | up |
| IBS-D.vs.Control | ERH | 2.856678 | 1.514338 | 0.00968023 | 1.220643 | up |
| IBS-D.vs.Control | Prostaglandin F2α-1-glyceryl ester | 2.25073 | 1.170393 | 0.010181876 | 1.833502 | up |
| IBS-D.vs.Control | LKK | 1.954838 | 0.967049 | 0.010290997 | 1.701844 | up |
| IBS-D.vs.Control | (1E)-5-hydroxy-1,7-diphenylhept-1-en-3-one | 1.619849 | 0.69586 | 0.010660637 | 1.368822 | up |
| IBS-D.vs.Control | 5α-Dihydrotestosterone | 0.29446 | -1.76386 | 0.011215812 | 1.877076 | down |
| IBS-D.vs.Control | Lysopc 16:1 | 2.846113 | 1.508993 | 0.011490029 | 1.728816 | up |
| IBS-D.vs.Control | Pipecolic acid | 0.6382 | -0.64792 | 0.011700885 | 1.608922 | down |
| IBS-D.vs.Control | N6-Acetyl-L-lysine | 1.817538 | 0.861985 | 0.012188241 | 1.993068 | up |
| IBS-D.vs.Control | Dehydroepiandrosterone | 0.33006 | -1.5992 | 0.012902895 | 1.689585 | down |
| IBS-D.vs.Control | Desoxycortone | 0.334501 | -1.57992 | 0.013160016 | 1.815351 | down |
| IBS-D.vs.Control | tert-butyl N-[2-(benzylamino)-1-methyl-2-oxoethyl]carbamate | 2.018954 | 1.013608 | 0.013540773 | 1.735477 | up |
| IBS-D.vs.Control | SM 9:1;2O/34:8 | 0.413449 | -1.27422 | 0.013637887 | 1.778821 | down |
| IBS-D.vs.Control | 2-methyl-N-[4-(4-methylpiperazino)benzyl]benzenesulfonamide | 0.620381 | -0.68877 | 0.014184217 | 1.644362 | down |
| IBS-D.vs.Control | N-{5-[(dimethylamino)sulfonyl]-2-methylphenyl}cyclohexanecarboxamide | 2.154955 | 1.107658 | 0.014850635 | 1.749856 | up |
| IBS-D.vs.Control | 4-(Diethylamino)salicylaldehyde | 0.579617 | -0.78683 | 0.014938559 | 1.801485 | down |
| IBS-D.vs.Control | Octhilinone | 0.251081 | -1.99377 | 0.015142415 | 2.026586 | down |
| IBS-D.vs.Control | Nonanoic acid | 2.980834 | 1.575716 | 0.016388714 | 1.802621 | up |
| IBS-D.vs.Control | XLR11 N-(4-hydroxypentyl) metabolite-d5 | 1.784902 | 0.835845 | 0.016530001 | 1.640276 | up |
| IBS-D.vs.Control | LPS 15:0 | 0.499062 | -1.00271 | 0.0170404 | 1.75265 | down |
| IBS-D.vs.Control | Ergocalciferol | 0.373796 | -1.41968 | 0.017962699 | 1.922175 | down |
| IBS-D.vs.Control | DPK | 2.724815 | 1.446158 | 0.01816998 | 1.768662 | up |
| IBS-D.vs.Control | 3-(2,6-difluorophenyl)-4-ethyl-1H-pyrazole | 0.653688 | -0.61333 | 0.018594747 | 1.534734 | down |
| IBS-D.vs.Control | Dl-Lanthionine | 2.325092 | 1.217288 | 0.018726574 | 1.824258 | up |
| IBS-D.vs.Control | 2,3-Dinor prostaglandin E1 | 3.380423 | 1.757204 | 0.019248179 | 1.680194 | up |
| IBS-D.vs.Control | O-Acetylserine | 1.743715 | 0.802164 | 0.019875517 | 1.582026 | up |
| IBS-D.vs.Control | DG O-16:3_28:7 | 0.359427 | -1.47623 | 0.020054968 | 1.38824 | down |
| IBS-D.vs.Control | MGMG (18:2) | 3.000286 | 1.5851 | 0.020089022 | 1.605828 | up |
| IBS-D.vs.Control | 13-Hpotre(R) | 2.889522 | 1.530831 | 0.02044705 | 1.769074 | up |
| IBS-D.vs.Control | 2-Aminoadipic acid | 2.091572 | 1.064588 | 0.020502075 | 1.701107 | up |
| IBS-D.vs.Control | LPE 14:0 | 0.584622 | -0.77443 | 0.022029162 | 1.821698 | down |
| IBS-D.vs.Control | Dehydroepiandrosterone (DHEA) | 0.457637 | -1.12773 | 0.022308239 | 1.670499 | down |
| IBS-D.vs.Control | EKK | 1.625721 | 0.70108 | 0.022311462 | 1.146065 | up |
| IBS-D.vs.Control | Sorbitan monopalmitate | 2.841233 | 1.506517 | 0.022733432 | 1.860622 | up |
| IBS-D.vs.Control | LPC O-16:0 | 0.315259 | -1.66539 | 0.022856402 | 1.571187 | down |
| IBS-D.vs.Control | MLK | 1.721712 | 0.783844 | 0.02295822 | 1.386894 | up |
| IBS-D.vs.Control | 6-(Dimethylamino)purine | 0.479531 | -1.0603 | 0.023066551 | 1.665552 | down |
| IBS-D.vs.Control | SPH | 4.045738 | 2.016403 | 0.02378882 | 1.516374 | up |
| IBS-D.vs.Control | FLK | 1.998908 | 0.999212 | 0.023994742 | 1.777883 | up |
| IBS-D.vs.Control | Ciprostene | 0.305513 | -1.7107 | 0.024595589 | 1.882275 | down |
| IBS-D.vs.Control | 4-Hydroxybenzoic acid | 0.424711 | -1.23545 | 0.024839047 | 1.737391 | down |
| IBS-D.vs.Control | Tetrahydroaldosterone | 3.4247 | 1.775977 | 0.025049754 | 1.778585 | up |
| IBS-D.vs.Control | Indoxylsulfuric acid | 0.64198 | -0.6394 | 0.025358701 | 1.731061 | down |
| IBS-D.vs.Control | N-Acetyl-DL-glutamic acid | 1.746788 | 0.804704 | 0.025741869 | 1.645045 | up |
| IBS-D.vs.Control | 16-Heptadecyne-1,2,4-triol | 0.617487 | -0.69552 | 0.02591935 | 1.819584 | down |
| IBS-D.vs.Control | Tramadol N-Oxide | 1.639178 | 0.712973 | 0.02595734 | 1.25406 | up |
| IBS-D.vs.Control | Kanosamine | 2.475978 | 1.307999 | 0.026257127 | 1.508705 | up |
| IBS-D.vs.Control | Phylloquinone | 0.30594 | -1.70868 | 0.026420631 | 1.851276 | down |
| IBS-D.vs.Control | L-Phenylalanine | 0.572541 | -0.80455 | 0.026625992 | 1.598738 | down |
| IBS-D.vs.Control | D-Phenylalanine | 0.528971 | -0.91874 | 0.026802117 | 1.475471 | down |
| IBS-D.vs.Control | Androsterone | 0.452616 | -1.14364 | 0.0276398 | 1.749402 | down |
| IBS-D.vs.Control | 1-(3-ethyl-2,4-dihydroxy-6-methoxyphenyl)butan-1-one | 0.483113 | -1.04957 | 0.027672381 | 1.735332 | down |
| IBS-D.vs.Control | TKK | 2.67484 | 1.419453 | 0.027845845 | 1.549416 | up |
| IBS-D.vs.Control | Nicotinuric Acid | 0.301119 | -1.7316 | 0.027953898 | 1.741301 | down |
| IBS-D.vs.Control | (+/-)5(6)-EET Ethanolamide | 0.386912 | -1.36992 | 0.028071353 | 1.511204 | down |
| IBS-D.vs.Control | (+/-)11(12)-DiHET | 0.422386 | -1.24336 | 0.028103428 | 1.605629 | down |
| IBS-D.vs.Control | 4-Hydroxymandelonitrile | 0.599949 | -0.73709 | 0.02816604 | 1.531238 | down |
| IBS-D.vs.Control | Capryloylglycine | 0.604353 | -0.72654 | 0.028395268 | 1.522014 | down |
| IBS-D.vs.Control | LPG 16:1 | 0.39974 | -1.32287 | 0.028445162 | 1.525803 | down |
| IBS-D.vs.Control | N-(4-methoxy-1-methyl-1H-indazol-3-yl)-5-methyl-3-isoxazolecarboxamide | 8.106851 | 3.019142 | 0.028855723 | 1.534573 | up |
| IBS-D.vs.Control | Paracetamol | 2.055197 | 1.039277 | 0.028968834 | 1.848758 | up |
| IBS-D.vs.Control | 8-(1,2-dihydroxy-3-methylbut-3-en-1-yl)-7-methoxy-2H-chromen-2-one | 2.327726 | 1.218921 | 0.02900511 | 1.778877 | up |
| IBS-D.vs.Control | cAMP | 2.022142 | 1.015884 | 0.02924323 | 1.649502 | up |
| IBS-D.vs.Control | Glutathione | 2.243045 | 1.165458 | 0.029479726 | 1.468683 | up |
| IBS-D.vs.Control | 5-Phenylvaleric Acid | 1.809054 | 0.855235 | 0.029593591 | 1.62709 | up |
| IBS-D.vs.Control | Resolvin E1 | 2.270577 | 1.183059 | 0.029646246 | 1.663903 | up |
| IBS-D.vs.Control | 5'-Adenylic acid | 2.019356 | 1.013895 | 0.029740303 | 1.651627 | up |
| IBS-D.vs.Control | 1,2-dihydroxyheptadec-16-yn-4-yl acetate | 0.475555 | -1.07232 | 0.029839742 | 1.772838 | down |
| IBS-D.vs.Control | TLK | 1.861444 | 0.896422 | 0.029875953 | 1.693539 | up |
| IBS-D.vs.Control | octadec-9-ynoic acid | 3.04916 | 1.608412 | 0.030001605 | 1.876421 | up |
| IBS-D.vs.Control | 13,14-Dihydro prostaglandin E1 | 1.835573 | 0.876231 | 0.030425635 | 1.726582 | up |
| IBS-D.vs.Control | 13,14-dihydro-15-keto Prostaglandin A2 | 0.576344 | -0.795 | 0.031108161 | 1.588724 | down |
| IBS-D.vs.Control | Equol | 2.508427 | 1.326783 | 0.031375769 | 1.685847 | up |
| IBS-D.vs.Control | Gibberellin A4 | 0.075715 | -3.72327 | 0.031850583 | 1.844272 | down |
| IBS-D.vs.Control | LMK | 2.332639 | 1.221963 | 0.031864637 | 1.603371 | up |
| IBS-D.vs.Control | PC 32:1 | 0.41285 | -1.27631 | 0.032010628 | 1.855612 | down |
| IBS-D.vs.Control | 2-[(3S)-1-(Cyclohexylmethyl)-3-pyrrolidinyl]-5-fluoro-1H-benzimidazole | 1.659122 | 0.73042 | 0.032052376 | 1.356135 | up |
| IBS-D.vs.Control | Testosterone sulfate | 0.07976 | -3.64819 | 0.032076875 | 1.899316 | down |
| IBS-D.vs.Control | L-Glutamic acid | 1.630711 | 0.705501 | 0.032115713 | 1.431056 | up |
| IBS-D.vs.Control | N-(4-chlorophenyl)-N'-cyclohexylthiourea | 0.436217 | -1.19688 | 0.033134245 | 1.207542 | down |
| IBS-D.vs.Control | D-2-Aminoadipic acid | 1.734227 | 0.794293 | 0.033483248 | 1.396389 | up |
| IBS-D.vs.Control | cis-4-Hydroxy-D-proline | 1.600122 | 0.678182 | 0.034531685 | 1.733827 | up |
| IBS-D.vs.Control | Hydroxyproline | 1.600122 | 0.678182 | 0.034531685 | 1.733827 | up |
| IBS-D.vs.Control | trans-3-Hexenoic acid | 1.513653 | 0.598035 | 0.034793894 | 1.641495 | up |
| IBS-D.vs.Control | LPC O-18:1 | 0.432724 | -1.20848 | 0.035185217 | 1.477968 | down |
| IBS-D.vs.Control | Cannabigerolic acid | 1.645263 | 0.718318 | 0.035861581 | 1.208943 | up |
| IBS-D.vs.Control | MGDG O-11:0_26:3 | 3.120104 | 1.641594 | 0.035873925 | 1.653715 | up |
| IBS-D.vs.Control | D-Turanose | 3.111867 | 1.63778 | 0.036876486 | 1.758044 | up |
| IBS-D.vs.Control | QLK | 1.531939 | 0.615359 | 0.037038938 | 1.128333 | up |
| IBS-D.vs.Control | Alanyltyrosine | 1.578058 | 0.65815 | 0.037408215 | 1.680576 | up |
| IBS-D.vs.Control | Prostaglandin B1 | 1.897476 | 0.924081 | 0.037557801 | 1.560663 | up |
| IBS-D.vs.Control | Taurocholic acid | 10.02597 | 3.32567 | 0.038392367 | 1.442852 | up |
| IBS-D.vs.Control | 11-Ketoetiocholanolone | 0.636309 | -0.6522 | 0.038432185 | 1.490049 | down |
| IBS-D.vs.Control | Hesperetin | 2.077397 | 1.054777 | 0.038735568 | 1.384378 | up |
| IBS-D.vs.Control | 4-Hydroxybenzaldehyde | 0.651872 | -0.61734 | 0.039217733 | 1.380147 | down |
| IBS-D.vs.Control | Lysope 16:0 | 0.461057 | -1.11698 | 0.039619779 | 1.659485 | down |
| IBS-D.vs.Control | 23-Norcholic acid | 2.43868 | 1.286101 | 0.040577446 | 1.622761 | up |
| IBS-D.vs.Control | 2-piperidinobenzoic acid | 0.564685 | -0.82448 | 0.041017424 | 1.602752 | down |
| IBS-D.vs.Control | ST 29:1;O;S | 2.070331 | 1.049861 | 0.04119262 | 1.717574 | up |
| IBS-D.vs.Control | Malonic acid | 2.236989 | 1.161558 | 0.041738995 | 1.667998 | up |
| IBS-D.vs.Control | Lithocholic Acid | 0.219429 | -2.18818 | 0.042075038 | 1.715475 | down |
| IBS-D.vs.Control | Phe-Phe | 2.387084 | 1.255249 | 0.042220736 | 1.727707 | up |
| IBS-D.vs.Control | 16,16-Dimethyl prostaglandin A1 | 0.228035 | -2.13267 | 0.042457212 | 1.867171 | down |
| IBS-D.vs.Control | 22(S)-Hydroxycholesterol | 0.479283 | -1.06105 | 0.042636292 | 1.650736 | down |
| IBS-D.vs.Control | 4-Aminohippuric acid | 0.592711 | -0.7546 | 0.044469305 | 1.713371 | down |
| IBS-D.vs.Control | Adrenosterone | 0.631392 | -0.66339 | 0.044503635 | 1.28318 | down |
| IBS-D.vs.Control | LPK | 1.584475 | 0.664004 | 0.045042286 | 1.276236 | up |
| IBS-D.vs.Control | Cyclohexylsulfamate | 2.688211 | 1.426646 | 0.045644208 | 1.447301 | up |
| IBS-D.vs.Control | L-Pyroglutamic acid | 2.331133 | 1.221031 | 0.045734133 | 1.896556 | up |
| IBS-D.vs.Control | APH | 2.846388 | 1.509132 | 0.045984653 | 1.490032 | up |
| IBS-D.vs.Control | 1,7-bis(4-hydroxyphenyl)heptan-3-one | 1.973961 | 0.981093 | 0.046424039 | 1.592985 | up |
| IBS-D.vs.Control | δ-Ribono-1,4-lactone | 1.675594 | 0.744673 | 0.046586065 | 1.175158 | up |
| IBS-D.vs.Control | LPE 15:0 | 0.42892 | -1.22122 | 0.046675014 | 1.777127 | down |
| IBS-D.vs.Control | Gly-Val | 0.381105 | -1.39174 | 0.046771874 | 1.481185 | down |
| IBS-D.vs.Control | Monolaurin | 4.486184 | 2.165489 | 0.04698261 | 1.65467 | up |
| IBS-D.vs.Control | Sodium cholate | 1.821148 | 0.864848 | 0.047240345 | 1.535374 | up |
| IBS-D.vs.Control | Gly-Tyr-Ala | 1.531928 | 0.615349 | 0.047860658 | 1.690613 | up |
| IBS-D.vs.Control | LPC 18:3-SN1 | 0.51534 | -0.9564 | 0.047899942 | 1.467548 | down |
| IBS-D.vs.Control | 2-Oxindole | 0.632584 | -0.66067 | 0.0480811 | 1.481731 | down |
| IBS-D.vs.Control | LPS 16:0 | 0.193737 | -2.36783 | 0.048250786 | 1.628604 | down |
| IBS-D.vs.Control | LPS 17:2 | 0.595132 | -0.74872 | 0.048309012 | 1.640589 | down |
| IBS-D.vs.Control | Homoarginine | 0.52235 | -0.93691 | 0.048370895 | 1.410569 | down |
| IBS-D.vs.Control | LPC 18:2-SN1 | 0.451036 | -1.14868 | 0.048566636 | 1.569722 | down |
| IBS-D.vs.Control | LPE 20:4 | 2.939395 | 1.555519 | 0.049064735 | 1.262835 | up |
| IBS-D.vs.Control | Glycocholic acid | 2.906142 | 1.539105 | 0.049762656 | 1.313836 | up |
| TXYF-H.vs.IBS-D | ethyl 1-(4-acetyl-2-aminophenyl)piperidine-4-carboxylate | 33.46034 | 5.06438 | 0.000161741 | 3.147124 | up |
| TXYF-H.vs.IBS-D | Royal jelly acid | 2.523148 | 1.335225 | 0.000350604 | 2.852772 | up |
| TXYF-H.vs.IBS-D | N-benzyl-N-isopropyl-N'-(4-isopropylphenyl)thiourea | 5.668883 | 2.503064 | 0.000512705 | 2.624351 | up |
| TXYF-H.vs.IBS-D | Valsartan metabolite | 2.235674 | 1.16071 | 0.001277869 | 2.84676 | up |
| TXYF-H.vs.IBS-D | mesaconic acid | 1.672757 | 0.742228 | 0.00185329 | 2.784386 | up |
| TXYF-H.vs.IBS-D | Tramadol N-Oxide | 1.763492 | 0.818435 | 0.002357749 | 1.7588 | up |
| TXYF-H.vs.IBS-D | (5E)-7-methylidene-10-oxo-4-(propan-2-yl)undec-5-enoic acid | 4.86051 | 2.281108 | 0.002749765 | 2.412791 | up |
| TXYF-H.vs.IBS-D | 4-Butylresorcinol | 3.092393 | 1.628724 | 0.003205839 | 2.815853 | up |
| TXYF-H.vs.IBS-D | Glu-Val-Phe | 0.425486 | -1.23282 | 0.003522496 | 2.709273 | down |
| TXYF-H.vs.IBS-D | LPE 15:0 | 1.697417 | 0.763341 | 0.003871849 | 1.656811 | up |
| TXYF-H.vs.IBS-D | 20-Hydroxy-(5Z,8Z,11Z,14Z)-eicosatetraenoic acid | 2.59585 | 1.376207 | 0.004577421 | 2.85356 | up |
| TXYF-H.vs.IBS-D | 8-iso-15-keto Prostaglandin F2α | 1.84101 | 0.880498 | 0.004640183 | 2.148136 | up |
| TXYF-H.vs.IBS-D | LPE 16:1 | 1.891121 | 0.919242 | 0.004862055 | 2.327273 | up |
| TXYF-H.vs.IBS-D | 3-[(4-chlorophenyl)thio]-1-(3-pyridylmethyl)pyrrolidine-2,5-dione | 0.216108 | -2.21018 | 0.005357765 | 2.704074 | down |
| TXYF-H.vs.IBS-D | Indole-3-acetic acid | 1.794954 | 0.843947 | 0.006326881 | 2.301878 | up |
| TXYF-H.vs.IBS-D | Pyroglutamic acid | 0.597509 | -0.74297 | 0.006379697 | 2.525762 | down |
| TXYF-H.vs.IBS-D | 2,3-Dinor prostaglandin E1 | 0.256594 | -1.96244 | 0.006736097 | 2.467489 | down |
| TXYF-H.vs.IBS-D | D-(-)-Glutamine | 0.563282 | -0.82807 | 0.007281709 | 1.693279 | down |
| TXYF-H.vs.IBS-D | 5-[(Benzoyloxy)methyl]-4,5,6-trihydroxy-2-cyclohexen-1-yl benzoate | 7.689375 | 2.942866 | 0.008644893 | 2.805857 | up |
| TXYF-H.vs.IBS-D | 13-Hpotre(R) | 0.325702 | -1.61837 | 0.008759709 | 2.310185 | down |
| TXYF-H.vs.IBS-D | O-Acetylserine | 0.50699 | -0.97997 | 0.009053972 | 2.399774 | down |
| TXYF-H.vs.IBS-D | Eicosapentaenoic acid ethyl ester | 1.69284 | 0.759446 | 0.009283197 | 1.620094 | up |
| TXYF-H.vs.IBS-D | Flavin Adenine Dinucleotide | 1.92574 | 0.945413 | 0.009292212 | 2.261922 | up |
| TXYF-H.vs.IBS-D | 6-methyl-7-nitro-2,3-dihydro-1,4-benzodioxine | 1.651735 | 0.723982 | 0.009495449 | 2.361179 | up |
| TXYF-H.vs.IBS-D | 2-(1H-indol-3-yl)acetic acid | 1.574957 | 0.655312 | 0.011407193 | 2.105308 | up |
| TXYF-H.vs.IBS-D | L-Aspartic acid | 0.517816 | -0.94949 | 0.013533619 | 2.293796 | down |
| TXYF-H.vs.IBS-D | Bilirubin | 0.299738 | -1.73823 | 0.01389534 | 2.114549 | down |
| TXYF-H.vs.IBS-D | 19(R)-hydroxy Prostaglandin A2 | 1.738818 | 0.798107 | 0.013965996 | 1.479806 | up |
| TXYF-H.vs.IBS-D | Threonine | 0.634154 | -0.6571 | 0.014075414 | 1.470261 | down |
| TXYF-H.vs.IBS-D | ERH | 3.204285 | 1.680002 | 0.014788859 | 1.690671 | up |
| TXYF-H.vs.IBS-D | Octadecanamine | 3.356732 | 1.747057 | 0.015369914 | 1.942465 | up |
| TXYF-H.vs.IBS-D | Υ-Aminobutyric acid (GABA) | 0.511813 | -0.96631 | 0.016110541 | 2.134272 | down |
| TXYF-H.vs.IBS-D | 22(S)-Hydroxycholesterol | 1.781646 | 0.833211 | 0.017219341 | 1.802062 | up |
| TXYF-H.vs.IBS-D | D-glutamine | 0.636769 | -0.65116 | 0.017757578 | 1.569266 | down |
| TXYF-H.vs.IBS-D | Ergosterol peroxide | 2.099425 | 1.069994 | 0.01795644 | 2.484626 | up |
| TXYF-H.vs.IBS-D | 2-chloro-4-(2-chloro-3,5-dinitrothien-4-yl)-3,5-dinitrothiophene | 6.891965 | 2.784915 | 0.018520481 | 2.683737 | up |
| TXYF-H.vs.IBS-D | L-Homocitrulline | 2.880165 | 1.526151 | 0.01878991 | 2.430682 | up |
| TXYF-H.vs.IBS-D | D-Glucuronic acid | 3.229893 | 1.691486 | 0.019412471 | 2.605217 | up |
| TXYF-H.vs.IBS-D | 3-Hydroxydecanoic acid | 1.627737 | 0.702868 | 0.021648891 | 2.374158 | up |
| TXYF-H.vs.IBS-D | 2-methyl-2,3,4,5-tetrahydro-1,5-benzoxazepin-4-one | 1.512024 | 0.596481 | 0.021905149 | 2.052132 | up |
| TXYF-H.vs.IBS-D | Serotonin | 1.525581 | 0.609359 | 0.023161576 | 2.330143 | up |
| TXYF-H.vs.IBS-D | N-(2,6-dimethylphenyl)-2-piperidinopropanamide | 0.604295 | -0.72667 | 0.024749624 | 1.821587 | down |
| TXYF-H.vs.IBS-D | Nicotinamide | 0.174223 | -2.52099 | 0.025766407 | 1.942775 | down |
| TXYF-H.vs.IBS-D | Tropine | 3.699515 | 1.887336 | 0.027550814 | 2.499326 | up |
| TXYF-H.vs.IBS-D | 1-(4-hydroxyphenyl)propane-1,2-diol | 2.172253 | 1.119192 | 0.027832232 | 2.142613 | up |
| TXYF-H.vs.IBS-D | D-2-Aminoadipic acid | 0.534896 | -0.90267 | 0.02918375 | 2.184299 | down |
| TXYF-H.vs.IBS-D | 11-Deoxy prostaglandin F1α | 1.502012 | 0.586896 | 0.033183859 | 1.952295 | up |
| TXYF-H.vs.IBS-D | 4-Hydroxymandelonitrile | 1.701272 | 0.766614 | 0.033212445 | 1.980312 | up |
| TXYF-H.vs.IBS-D | Arachidonic acid methyl ester | 2.008202 | 1.005904 | 0.033723698 | 1.566807 | up |
| TXYF-H.vs.IBS-D | 4-(3,4-dihydro-2H-1,5-benzodioxepin-7-ylamino)-4-oxobutanoic acid | 0.165373 | -2.5962 | 0.034993565 | 1.890768 | down |
| TXYF-H.vs.IBS-D | L-Glutamic acid | 0.581288 | -0.78267 | 0.035535238 | 2.171853 | down |
| TXYF-H.vs.IBS-D | 2-Aminoadipic acid | 0.533983 | -0.90513 | 0.036474437 | 1.843297 | down |
| TXYF-H.vs.IBS-D | LPE 14:0 | 1.514176 | 0.598533 | 0.03655888 | 1.890538 | up |
| TXYF-H.vs.IBS-D | 6-Methylquinoline | 2.012023 | 1.008647 | 0.038652169 | 2.268659 | up |
| TXYF-H.vs.IBS-D | Tryptamine | 2.012023 | 1.008647 | 0.038652169 | 2.268659 | up |
| TXYF-H.vs.IBS-D | 5α-Dihydrotestosterone | 2.394164 | 1.259522 | 0.041153239 | 1.714353 | up |
| TXYF-H.vs.IBS-D | 5-(benzyloxy)-2-(hydroxymethyl)-1,4-dihydropyridin-4-one | 1.822581 | 0.865983 | 0.042018839 | 1.760542 | up |
| TXYF-H.vs.IBS-D | LPS 16:1 | 2.580716 | 1.367771 | 0.042094041 | 2.187996 | up |
| TXYF-H.vs.IBS-D | N-Acetyl-aspartic acid | 0.575881 | -0.79616 | 0.042146464 | 1.694351 | down |
| TXYF-H.vs.IBS-D | gamma-Glutamylcysteine | 1.72117 | 0.783389 | 0.042153073 | 1.7486 | up |
| TXYF-H.vs.IBS-D | gamma-Glutamylleucine | 0.58664 | -0.76945 | 0.042630508 | 1.598806 | down |
| TXYF-H.vs.IBS-D | 6-(7-methyloctyl)-1H,3H,4H,6H-furo[3,4-c]furan-1-one | 2.570958 | 1.362306 | 0.042860372 | 2.203937 | up |
| TXYF-H.vs.IBS-D | XLR11 N-(4-hydroxypentyl) metabolite-d5 | 0.597316 | -0.74343 | 0.043078109 | 1.959101 | down |
| TXYF-H.vs.IBS-D | ST 24:1;O2;G | 0.30858 | -1.69628 | 0.043748992 | 1.870054 | down |
| TXYF-H.vs.IBS-D | Citrulline | 0.539178 | -0.89117 | 0.043812775 | 1.878127 | down |
| TXYF-H.vs.IBS-D | L-(+)-Citrulline | 0.642992 | -0.63713 | 0.043943063 | 2.108559 | down |
| TXYF-H.vs.IBS-D | Sodium cholate | 0.544432 | -0.87718 | 0.044063965 | 1.984805 | down |
| TXYF-H.vs.IBS-D | Saccharin | 1.725503 | 0.787017 | 0.044266067 | 1.390895 | up |
| TXYF-H.vs.IBS-D | U-47700-d6 | 2.383523 | 1.253096 | 0.044761588 | 1.909362 | up |
| TXYF-H.vs.IBS-D | 2-[2-(3,4-dichloro-2-cyanophenyl)hydrazono]malononitrile | 2.341187 | 1.22724 | 0.046041457 | 2.146691 | up |
| TXYF-H.vs.IBS-D | Pipecolic acid | 1.586131 | 0.665511 | 0.046098914 | 1.950404 | up |
| TXYF-H.vs.IBS-D | Cytosine | 0.629236 | -0.66833 | 0.046566501 | 1.868201 | down |
| TXYF-H.vs.IBS-D | 5-Hydroxyindole-3-acetic acid | 1.639226 | 0.713015 | 0.047777493 | 1.976985 | up |
| TXYF-H.vs.IBS-D | ethyl 3-[(4-chlorophenethyl)amino]-2-cyanoacrylate | 1.979691 | 0.985276 | 0.04881668 | 2.034638 | up |
